# Redox Epiphospholipidome in Programmed Cell Death Signaling: Catalytic Mechanisms and Regulation

**DOI:** 10.3389/fendo.2020.628079

**Published:** 2021-02-19

**Authors:** Valerian E. Kagan, Yulia Y. Tyurina, Irina I. Vlasova, Alexander A. Kapralov, Andrew A. Amoscato, Tamil S. Anthonymuthu, Vladimir A. Tyurin, Indira H. Shrivastava, Fatma B. Cinemre, Andrew Lamade, Michael W. Epperly, Joel S. Greenberger, Donald H. Beezhold, Rama K. Mallampalli, Apurva K. Srivastava, Hulya Bayir, Anna A. Shvedova

**Affiliations:** ^1^ Center for Free Radical and Antioxidant Health, Department of Environmental and Occupational Health, University of Pittsburgh, Pittsburgh, PA, United States; ^2^ World-Class Research Center “Digital Biodesign and Personalized Healthcare”, Sechenov First Moscow State Medical University, Moscow, Russia; ^3^ Department of Critical Care Medicine, Safar Center for Resuscitation Research, Children’s Neuroscience Institute, University of Pittsburgh, Pittsburgh, PA, United States; ^4^ Office of the Director, Health Effects Laboratory Division, NIOSH/CDC, Morgantown, WV, United States; ^5^ Department of Radiation Oncology, University of Pittsburgh, Pittsburgh, PA, United States; ^6^ Department of Internal Medicine, The Ohio State University, Columbus, OH, United States; ^7^ Laboratory of Human Toxicology and Pharmacology, Applied/Developmental Research Directorate, Leidos Biomedical Research, Inc., Frederick National Laboratory for Cancer Research, Frederick, MD, United States; ^8^ Exposure Assessment Branch, The National Institute for Occupational Safety and Health/Centers for Disease Control and Prevention (NIOSH/CDC), Morgantown, WV, United States

**Keywords:** regulated cell death, apoptosis, ferroptosis, phospholipid peroxidation, redox lipidomics, cytochrome *c*, cardiolipin, lipoxygenase

## Abstract

A huge diversification of phospholipids, forming the aqueous interfaces of all biomembranes, cannot be accommodated within a simple concept of their role as membrane building blocks. Indeed, a number of signaling functions of (phospho)lipid molecules has been discovered. Among these signaling lipids, a particular group of oxygenated polyunsaturated fatty acids (PUFA), so called lipid mediators, has been thoroughly investigated over several decades. This group includes oxygenated octadecanoids, eicosanoids, and docosanoids and includes several hundreds of individual species. Oxygenation of PUFA can occur when they are esterified into major classes of phospholipids. Initially, these events have been associated with non-specific oxidative injury of biomembranes. An alternative concept is that these post-synthetically oxidatively modified phospholipids and their adducts with proteins are a part of a redox epiphospholipidome that represents a rich and versatile language for intra- and inter-cellular communications. The redox epiphospholipidome may include hundreds of thousands of individual molecular species acting as meaningful biological signals. This review describes the signaling role of oxygenated phospholipids in programs of regulated cell death. Although phospholipid peroxidation has been associated with almost all known cell death programs, we chose to discuss enzymatic pathways activated during apoptosis and ferroptosis and leading to peroxidation of two phospholipid classes, cardiolipins (CLs) and phosphatidylethanolamines (PEs). This is based on the available LC-MS identification and quantitative information on the respective peroxidation products of CLs and PEs. We focused on molecular mechanisms through which two proteins, a mitochondrial hemoprotein cytochrome *c* (cyt *c*), and non-heme Fe lipoxygenase (LOX), change their catalytic properties to fulfill new functions of generating oxygenated CL and PE species. Given the high selectivity and specificity of CL and PE peroxidation we argue that enzymatic reactions catalyzed by cyt *c*/CL complexes and 15-lipoxygenase/phosphatidylethanolamine binding protein 1 (15LOX/PEBP1) complexes dominate, at least during the initiation stage of peroxidation, in apoptosis and ferroptosis. We contrast cell-autonomous nature of CLox signaling in apoptosis correlating with its anti-inflammatory functions *vs.* non-cell-autonomous ferroptotic signaling facilitating pro-inflammatory (necro-inflammatory) responses. Finally, we propose that small molecule mechanism-based regulators of enzymatic phospholipid peroxidation may lead to highly specific anti-apoptotic and anti-ferroptotic therapeutic modalities.

*The real reason for not committing suicide is because you always know how swell life gets again after the hell is over. **Ernest Hemingway***

*If you’re going through hell, keep going. **Winston Churchill***

## Signaling by Polyunsaturated Lipids: Autocrine, Paracrine, and Endocrine Types

Billions of years of evolution created and optimized mechanisms for efficient translation of genomic information into thousands of finely tuned protein machines (1) and perfected functional interactions between the proteins through sophisticated multi-leveled signaling systems (2). Lipids of biological membranes constitute a critical part of this complicated signaling network (3). The metabolic coordination requires that the flow of signaling information proceeds with optimized levels of fidelity and speed. Conservative estimates indicate that the number of proteins in the human proteome is on the order of 10^5^-10^6^ (4, 5). Thus it is not surprising that the diversity of signaling lipids coordinating multiple protein-protein and lipid-protein interactions within and between subcellular organelles, cells, and tissues may be even greater resulting in the possible >10^6^ of individual molecular species in the lipidome. Engagement of membrane phospholipids (PLs) in the signaling process occurs *via* their biochemical modifications leading to the appearance of small amounts of “unusual” PL molecules such as their hydrolysis or peroxidation products (6). This review is focused on oxidatively modified (phospho)lipids as the signaling entities. Among them are well known lipid mediators represented by oxygenated free polyunsaturated fatty acids (PUFA) as well as oxygenated PUFA esterified into different classes of membrane phospholipids (PLs). The latter group will be the subject of the current review.

Cultural beliefs of successful societies have led to the common opinion that suicidal elimination is not the necessary way to resolve life conflicts that may encompass transient dark episodes within an otherwise bright present and even more wonderful future. This optimistic view has been expressed in many statements by politicians, writers and other artistic celebrities (including those by W. Churchill and E. Hemingway quoted above). On the molecular level, however, the ruthlessness of life/death elimination decisions is frequently a necessary attribute of the high fidelity of cell populations and their adaptive adjustments. The suicidal programs of cell death are genetically pre-determined and deciphering their specific mechanisms represents one of the emerging fields of cell biology. The lipid-derived signals may act within a given cell (autocrine signaling), affect cells within the surrounding neighborhood (paracrine signaling) or act on remote targets using the circulatory system for the transportation of death signals (endocrine signaling). Currently, more than a dozen regulated death programs have been identified in cells that accumulate excessive amounts of the (geno)toxic materials and hence are recognized by the surveillance machinery as irreparably damaged. It is believed, but not proven, that peroxidation of polyunsaturated lipids (PUFA-lipids) has been associated with the initiation and execution of many, if not all, of these programs (7). In spite of these general associations, neither the specific roles of peroxidized PLs in the fulfillment of the programs nor their chemical identity have been identified. Notable exceptions are apoptosis and ferroptosis, two programs for which the progress in redox lipidomics has resulted in the deciphering of death signals.

## Redox Death Signals in Apoptosis and Ferroptosis

The structural core of biological membranes is formed by the bilayer of PLs—amphipathic molecules with long lipophilic hydrocarbon chains and water-soluble polar heads. The hydrophobic/hydrophilic balance of phospholipids dictates the organization of the bilayer in which lipophilic chains interact with each other while the polar head-groups are localized at interface with the aqueous phase. In PLs, the chains represent fatty acids covalently attached to two sites of the glycerol backbone whereas the third position is occupied by phospho-base that may be a non-charged zwitter-ion (when the negative charge of the phosphate is compensated by a positive charge) or carry a negative charge (when an extra negatively charged group is present). Fatty acyls of PLs may have no double bonds (saturated) or contain one (mono-unsaturated) or several methylene-interrupted double bonds [polyunsaturated PLs (PUFA-PL)]. While unsaturated PLs may be biosynthesized both in anaerobic and aerobic conditions, the huge diversity of PUFA-PLs is characteristic for different domains of aerobic life (8).

One of the most popular concepts explaining the presence of diversified PUFA-PLs in the lipid bilayer relates to their function as a regulator of membrane fluidity necessary for the rapid diffusion and conformational flexibility of membrane proteins (9). In spite of its attractive simplicity, this concept does not explain the huge molecular variety of PUFA-PLs. Indeed, contemporary lipidomics detects 10^3^–10^4^ individual molecular species of major classes of phospholipids in cells and tissues. This is a conservative estimate of the species with differing masses. Indeed, with the two acyls/PL molecule and a menu of >30 commonly found fatty acids the number of possible isomeric species of “two-legged” PLs should be close to 10^3^. However, for “three-legged” tri-glycerides this estimate would yield 10^4^ species and for “four-legged” mitochondrial cardiolipins (CL) – >10^5^ molecular species.

One of the prominent features of PUFA-PLs is their susceptibility to peroxidation *via* free radical mechanisms (10). These mechanisms may be comprised of enzymatic systems for the activation of oxygen and/or lipid substrates or occur non-enzymatically (see below). As the general schema of peroxidation includes abstraction of hydrogen from bis-allylic positions, PLs with multiple (four-six) double-bonds, are preferred substrates, particularly for non-enzymatic free radical reactions (10, 11). The primary product of the peroxidation process generates hydroperoxy-PLs (HOO-PLs). These products are not stable and readily undergo secondary decay reactions leading to a variety of electrophilic aldehydic-, keto-, hydroxy-derivatives as well as cyclic compounds (10). In terms of lipid diversification, this adds another order of magnitude to the possible number of individual phospholipids, thus bringing it to 10^5^ species. It should be noted, however, that the measurable amounts of peroxidized PLs in healthy cells and tissues is markedly lower than their non-oxidized parent PLs (12). This is partly due to the fact that only a fraction of PUFA-PLs are involved in peroxidation and also to the high reactivity of the secondary electrophilic decay products toward nucleophilic sites in proteins (13). As a result, the life-time of these products in “free” form may be relatively short as they form lipid-protein adducts. However, the levels of these products may increase many-fold in conditions associated with cell injury and death. These reactive secondary intermediates of PUFA-PL peroxidation represent the proximate entities affecting functions of numerous proteins (14). Given that the formation of these adducts is, in a way, a reaction of protein “lipidation” that may dramatically change the distribution and functional characteristics of the affected proteins, it has been hypothesized, although not proven, that electrophilic products of PL peroxidation and their adducts with specific proteins represent the proximate “gateways” of cell’s demise in regulated cell death programs.

## Types of Regulated Cell Death: Involvement of Lipid Peroxidation and Possible Involvement of PL-OOH

Since the time of the first detailed description of regulated cell death almost five decades ago, about a dozen different programs have been identified (15). The best described programs include apoptosis, necroptosis, pyroptosis, ferroptosis, entotic cell death, netotic cell death, parthanatos, lysosome-dependent cell death, autophagy-dependent cell death, alkaliptosis, and oxeiptosis (15). The majority of them have been qualified as responses to different types of stresses causing irreversible changes not only to one particular cell but also representing a high-risk threat to the entire community of surrounding cells. Among the different causative factors, oxidative stress has been universally identified as one of the leading mechanisms engaged early at the initiation or later during the execution stages of the death programs (15) (**Table 1**).

**Table 1:** Involvement of lipid peroxidation in the execution of regulated cell death programs.

Death type	Target	Stimuli	Lipid peroxidation	
			**Implicated but not evidenced by LC/MS (References)**	**Implicated with evidence by LC/MS (References)**
Apoptosis	Intrinsic pathway	STS; rotenone; ActD; Hyperoxia; NAO/light; g-IR; TBI; stretch		Kagan et al. (16); Tyurin et al. (17); Tyurina et al. (18, 19); Huang et al. (20); Mao et al. (21); Belikova et al. (22); Bayir et al. (23); Ji et al. (24)
	Extrinsic pathway	Anti-Fas	Jiang et al. (25); Serinkan et al. (26) (evidence by HPLC)	Wiernicki et al. (7)
Ferroptosis	System $X_{c}^{-}$	Erastin; IKE; sorafenib	Dixon et al. (27); Yang et al. (28); Larraufie et al. (29); Louandre et al. (30)	Gaschler et al. (31)
	GPX4	RSL3; with aferin A; FINO_2_; ML162; Smoke/COPD; BAY-87-2243	Dixon et al. (27); Yang et al. (32); Basit et al. (33)	Kagan et al. (34); Doll et al. (35); Wenzel et al. (36); Kapralov et al. (37); Dar et al. (38); Hassannia et al. (39); Gaschler et al. (31); Yoshida et al. (40); Wiernicki et al. (7)
	Glutamate-cysteine ligase	BSO	Yang et al. (32)	
	Glutathione-S-transferase	Artesunate	Eling et al. (41); Lisewski et al. (42)	
	FSP1	FSP1ko/RSL3	Bersuker et al. (43); Doll et al. (44)	
	iNOS	iNOS kd/RSL3		Kapralov et al. (37)
	Iron oxidation	FINO_2_		Gaschler et al. (31)
	Other	TBI; P. aeruginosa; viral infection; AKI; heart transplants	Matsushita et al. (45)	Kenny et al. (46); Wenzel et al. (36); Dar et al. (38); Li et al. (47)
Necroptosis	GSH depletion	Hemin; gallic acid	Laird et al. (48); Chung et al. (49)	
		Myocardial infarction (RIP3)	Ghardashi Afousi et al. (50)	
	GPX4	GPX4 ko	Canli et al. (51)	
		TNF-α		Wiernicki et al. (7)
Pyroptosis	Caspase-11	Gasdermin-D	Kang et al. (52); Chen et al. (53)	
	Other	HIRI; CI; LPS/ATP; sevoflurane	Zhang et al. (54); Liang et al. (55); Li et al. (56)	Wiernicki et al. (7)

*γ*-Irradiation, *γ*-IR; staurosporine, STS; actinomycin D, ActD; nonyl-acridine orange, NAO; acute kidney injury, AKI; buthionine sulfoximine, BSO; imidazole ketone erastin, IKE; traumatic brain injury, TBI; hepatic ischemia-reperfusion injury, HIRI; cerebral ischemia, CI.

Given the vague definition of what exactly “oxidative stress” means, attempts have been made to connect the death programs with specific pathways and manifestations of the aberrant redox metabolism. Due to the high sensitivity of PUFA-PL to oxidative modifications, lipid peroxidation (LPO) has been considered as one of the common denominators of programmed cell death (57, 58). However, the specific role and mechanisms of LPO in the pathways leading to cell demise remain poorly defined. This is due, to a large extent, to difficulties in the analysis of highly diversified and very low abundance LPO products (12). These technological problems were resolved with the advent of high-resolution liquid-chromatography-mass spectrometry (LC-MS) based redox lipidomics (6, 12) with its capability to detect, identify and quantitatively characterize a variety of PL oxidation products. While the application of this technology may provide important information in any of the known death pathways, so far the significant results have been obtained mostly for two death programs—apoptosis and ferroptosis (16, 21, 34, 36).

**Figure 1:**
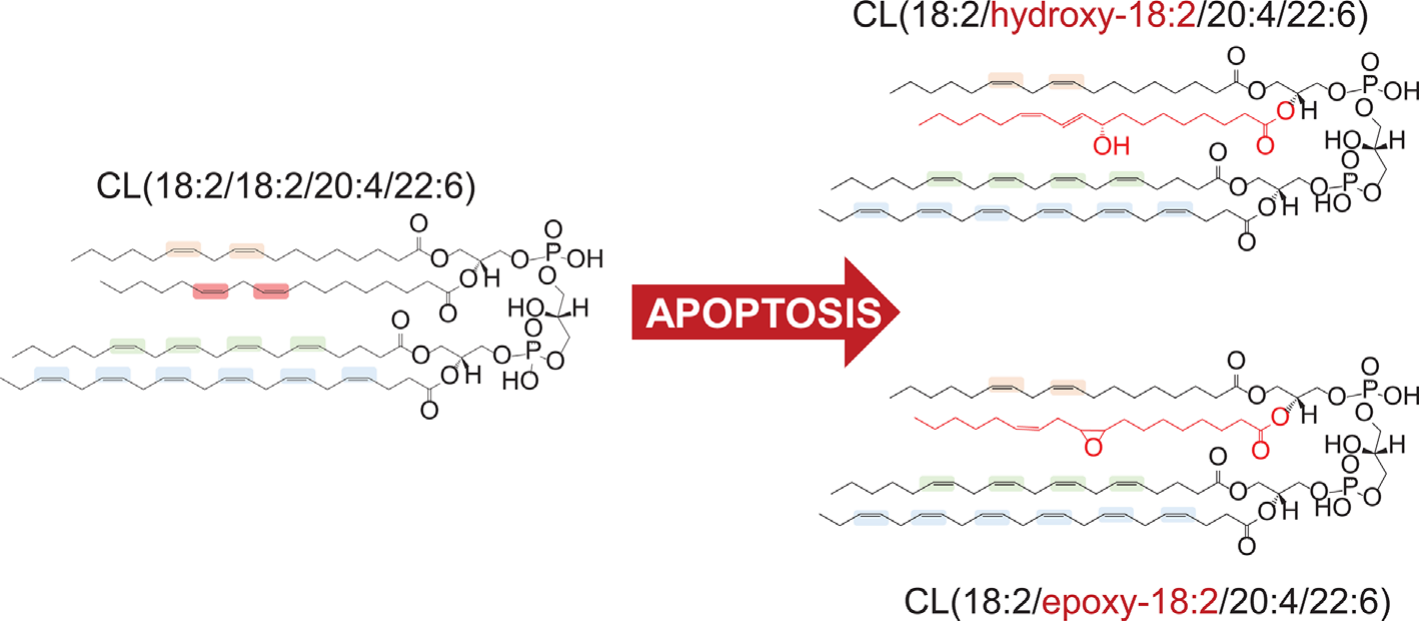
Preferential peroxidation of C18:2 residue in hetero-acylated cardiolipin (CL) molecule in apoptosis.

Numerous studies of PL peroxidation in model chemical and biochemical systems using different initiating agents (e.g., azo-initiators of peroxyl radicals, Fe-ascorbate dependent generators of HO• radicals) demonstrated that the susceptibility to oxidative modification is largely defined by the number of double bonds in the fatty acid residues (59). As a result, PLs with hexa-, penta-, and tetra-enoyl residues are the predominant peroxidation substrates as compared to doubly or triply unsaturated PLs. Notably, the nature of the polar part of the PL molecules was not influential as a factor determining vulnerability to oxidation. In sharp contrast, redox lipidomics studies of programmed death associated peroxidation has determined that there is a high selectivity of the process toward specific classes of PLs. Execution of the intrinsic apoptotic program revealed a high selectivity toward peroxidation of mitochondrial CLs whereby the species with C18:2 represented the major substrates (16, 21). Notably, CL molecular species containing more PUFA residues remained non-oxidized. Moreover, in hetero-acylated CL species, oxidation of C18:2 residues occurred preferentially even when C22:5 and C22:6 residues remained non-oxidized within the same molecule (**Figure 1**).

Ferroptosis-associated LPO was also highly selective toward a specific PL class—PUFA-containing phosphatidylethanolamines (PE) (34). Interestingly, two types of PE-molecular species with C20:4 and C22:4 displayed the highest sensitivity toward oxidative modification. In terms of positional specificity, the 15^th^ position in C20:4 and the 17^th^ position in C22:4 were the preferred oxidation sites. Importantly, these PE oxidation products exerted predictive features of ferroptosis biomarkers and displayed pro-ferroptotic activity upon co-incubations with target cells (34). A recent study demonstrated that not only di-acyl-phospholipids but also PUFA-plasmalogens (ether-phospholipids), synthesized in peroxisomes, underwent peroxidation in ferroptosis (60). Downregulation of ether phospholipids was associated with the increased resistance of cancer (carcinoma) cells to ferroptosis *in vivo*.

**Figure 2:**
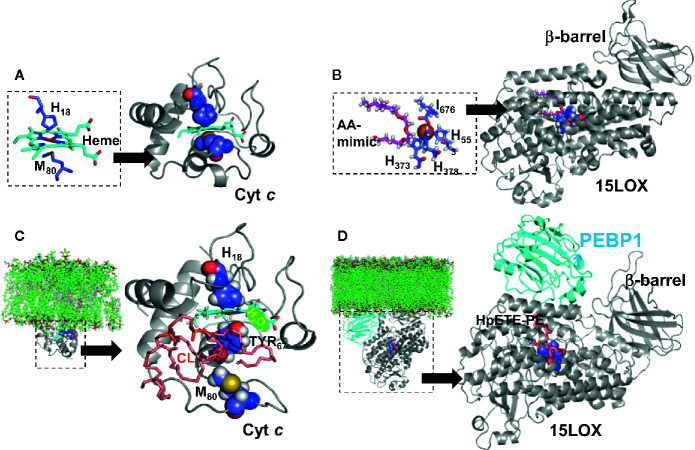
Structural models of phospholipid peroxidizing Fe-proteins. **(A)** Structure of cytochrome *c* (pdb ID 1hrc) (61) shown in ribbon diagram, with the heme molecule (in cyan) and the Cyt C residues (H18 and M80) highlighted in space filling representation. The inset shows the coordination of the Heme molecule by the Cyt C residues, including H18 and M80. **(B)** Structure of 15LOX Ipdb ID 4nre) (62), also shown in ribbon diagram, with the arachidonic mimic (AA) shown in magenta, stick representation. The catalytic site region is shown in detain in the inset. The b-barrel, which interfaces with the membrane, is labelled. **(C)** Structure of cyt *c*-cardiolipin complex. The residue M80 which coordinates the heme has moved away from the Heme molecule, leading to an unfolded cyt *c* conformation. This unfolded conformation, which was obtained from an earlier study (16) was used to dock a cardiolipin molecule (shown in pink). **(D)** Structure of 15LOX/PEBP1-HpETE-PE complex. The model of the complex, proposed by us (36) is shown in ribbon diagram. The PEBP1 (shown in cyan) is docked onto the 15LOX, and this complex model, was used to dock HpETE-PE molecule (shown in pink). The ligand docking for both cyt *c* and 15LOX/PEBP1 complex was performed by SMINA (63). The insets in **(C, D)** depict the interfacing of peroxidase complex and membrane bilayer. The models for the protein-membrane complexes were built using the Orientation of Proteins in Membrane webserver (https://opm.phar.umich.edu/ppm_server) which calculates translational and rotational position of membranes and proteins from their three-dimensional structures.

Selectivity and specificity of the PL peroxidation process in two different cases of apoptotic and ferroptotic (non-apoptotic) regulated cell death suggest a possible involvement of enzymatic catalytic mechanisms. Indeed, two different metalloproteins, a hemoprotein cytochrome *c* (cyt *c*) (**Figure 2A**) and a non-heme Fe-protein, 15-lipoxygenase (15LOX) (**Figure 2B**), have been identified as the highly likely enzymes initiating the peroxidation process in apoptosis and ferroptosis, respectively (16, 34). In both cases, the selectivity of the enzymatic peroxidation mechanisms is achieved due to the formation of lipid-protein or protein-protein complexes as described below.

## Enzymatic and Non-Enzymatic Lipid Peroxidation Mechanisms; Catalytic Role of Iron

**Figure 3:**
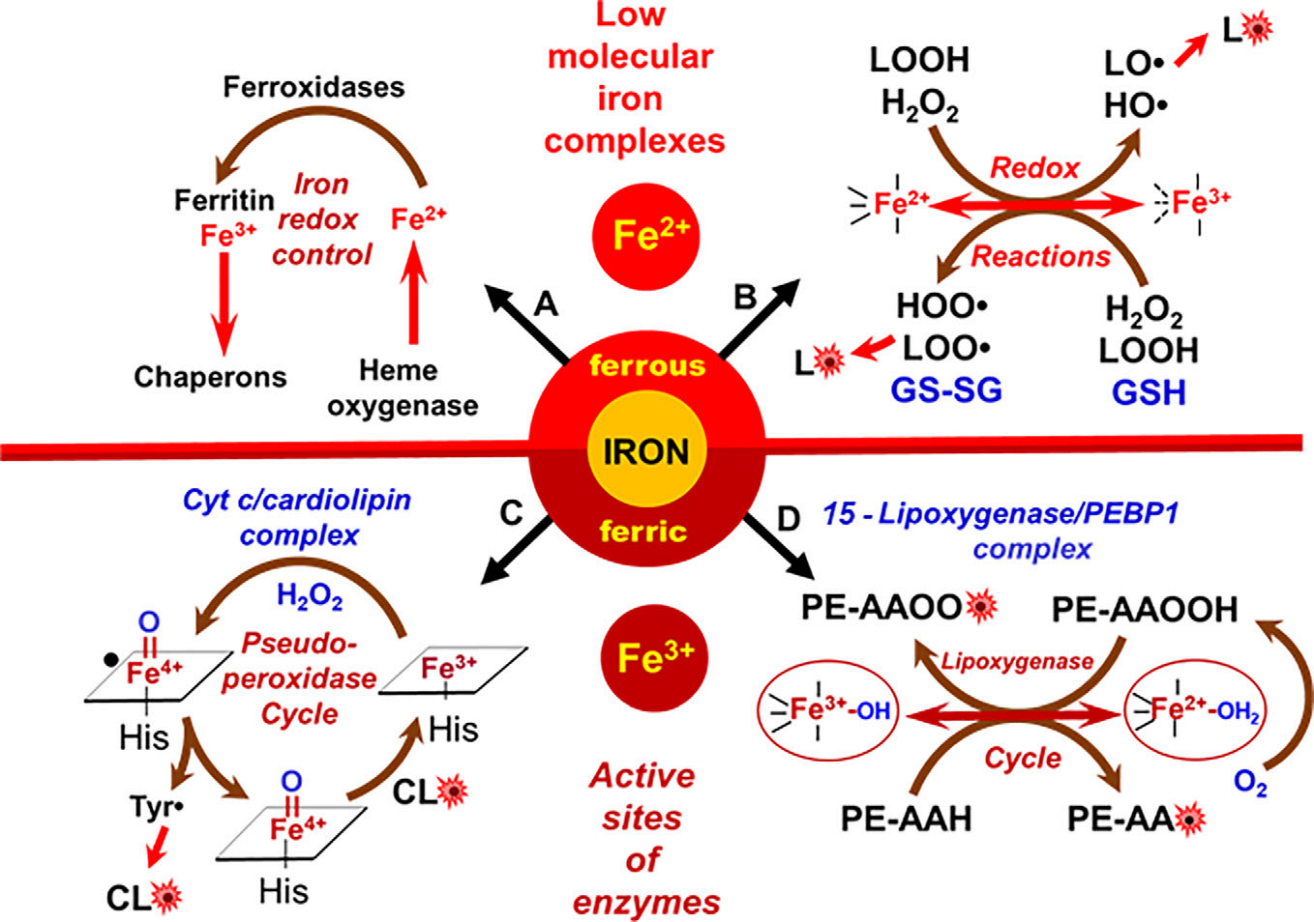
Metabolic redox pathways of iron in cells. **(A)** Tight control of redox-active iron in cells prevents its participation in peroxidation reactions; **(B)** redox activity of low molecular iron complexes (labile iron); **(C)** oxidation of CL in cyt *c*/CL complexes; **(D)** formation of lipid radicals by the catalytic site of 15LOX/PEBP1 complex.

PUFA residues of lipids are believed to be highly susceptible to oxidative modification by oxygen (10, 64). This process, LPO, proceeds *via* the formation of radical intermediates. The rate limiting stage is the initial formation of radicals that can further propagate the overall process. Therefore, the peroxidation rate is very low in the absence of catalysts. While there are many different radical initiators—physical factors like irradiation or chemical agents, like compounds spontaneously decomposing to form carbon-centered radicals—the most important biological peroxidation catalysts are transition metals, particularly iron (Fe) (65). Therefore, the levels of redox active free Fe-ions or “loosely bound” Fe in low molecular weight complexes are strictly controlled in cells and biological fluids (**Figure 3A**). Catalytic Fe of active enzymes is regulated by the protein structure. Fe required for these catalytic functions is delivered to the respective protein clients—Fe-sulfur proteins, hemoproteins, and non-heme Fe-proteins—by several specialized protein chaperons (66). Quantitatively, hemoproteins represent the most abundant endogenous source of Fe and catabolic degradation of these proteins accompanied by the release of ferrous ions (Fe^2+^) is operated by heme oxygenases (67). Specialized ferroxidases convert (Fe^2+^) to (Fe^3+^)—the form suitable for the intracellular iron storage by ferritin (**Figure 3**).

Normally regulated Fe-catalyzed reactions are enzymatic. However, under special conditions such as Fe-overload, excessive catabolism of Fe-proteins or diseases, aberrant non-enzymatic Fe-driven lipid peroxidation by poorly controlled low molecular Fe-complexes may occur, becoming excessive or even overwhelming (68). These reactions commonly proceed *via* the production of reactive oxygen species (ROS), particularly HO• radicals, generated in Fenton/Haber-Weiss reactions or *via* the formation of lipid radicals generated during decomposition of organic hydro peroxides, including lipid hydroperoxides (69) (**Figure 3B**). As cell death programs are based on regulated mechanisms, the associated LPO is initiated by a selective and specific enzymatic process (**Figures 3C, D**). Interestingly, the enzymes involved in the production of lipid death signals *via* peroxidation mechanisms during apoptosis and ferroptosis—cyt *c* and 15LOX—usually are involved in different biological functions. For example, cyt *c* is shuttling electrons between mitochondrial respiratory complexes III and IV in the intermembrane space (70). 15LOX is a dioxygenase catalyzing the formation of oxygenated lipid mediators from free PUFA, particularly free arachidonic acid [AA or eicosatetraenoic (ETE) acid 20:4] (71). Upon the initiation of the cell death program, these enzymes change their properties/functions and switch their activity to the peroxidation of PLs. The transformation of enzymatic activity occurs due to protein interaction with other molecules—a mitochondria-specific PL molecule, CL, in the case of cyt *c* (**Figure 2C**), and the protein PEBP1 in the case of 15LOX (**Figure 2D**).

### Cyt *c*-Catalyzed Peroxidation of Cardiolipins

Cyt *c* is a small mitochondrial intermembrane space hemoprotein (MW about 12.5 kD, 104 amino acids) (72). As a transporter of electrons, cyt *c* utilizes hexa-coordinated heme whereby the Fe has four coordination bonds with a protoporphyrin IX and His_18_ and Met_80_ at the proximal and distal sides as the fifth and sixth iron ligands (73). Participation in the execution of the apoptotic death program is a recently established important function of cyt *c*. There are two pro-apoptotic processes that depend on cyt *c*: i) apoptosome formation and ii) CL peroxidation (16, 74, 75). These two seemingly unrelated roles of cyt *c* may, in fact, be closely linked to each other. Cyt *c* that is released from mitochondria into the cytosol interacts with the apoptotic protease-activating factor 1 (Apaf-1) to form the apoptosome, thus initiating the activation of caspase-9 and downstream caspases (75). The outer mitochondrial membrane (OMM) is permeable to small molecules—co-factors, small peptides, etc.—that get readily released through the pores with the size limit of ~2nm in diameter (76). As the average diameter of the cyt *c* globule is ~4nm, it is normally retained within the intermembrane space (77). It has been hypothesized that products of CL peroxidation may accumulate in the OMM where they can get oxidized and became involved in the production of pores with diameters >4nm that will facilitate the release of cyt *c* from the intermembrane space into the cytosol.

The accumulation of irreparable defects in mitochondria triggers their elimination through a special type of autophagy, mitophagy (78). This is a multistage process in which the signaling by a mitochondria-specific CL plays a prominent role. Normally confined almost exclusively to the matrix leaflet of the inner mitochondria membrane (IMM), CL undergoes several trans-membrane migrations to the mitochondrial surface (the outer leaflet of the OMM) (79, 80). Externalized CL binds microtubule-associated proteins 1A/1B light chain 3B (LC3) (79), one of the central executioners of autophagy. Timely and successful elimination of damaged organelles *via* activation of mitophagy is a pro-survival mechanism (81). However, incomplete autophageal digestion of injured mitochondria with dysregulated electron transport represents a peril to the entire community of surrounding cells. As a result, the entire cell undergoes apoptotic elimination.

The transition from the pro-survival mitophagic to the apoptotic death program relies on a new interaction of cyt *c* with CL (which becomes possible during CL migration from IMM to OMM). Noteworthy, formation of the cyt *c*/CL complex causes a strong negative shift of its redox potential (by ~400 mV) such that cyt *c* can no longer act as an electron acceptor from complex III, and function as an electron carrier in the respiratory chain (82). This results in the elevated production of superoxide anion-radicals and their dismutation to H_2_O_2_. The latter can be used as a source of oxidizing equivalents for a peroxidase reaction, provided that an inadvertent peroxidase activity is present in the microenvironment. The formation of cyt *c*/CL complexes offers this opportunity.

Evidently both pools of CL facing the intermembrane space—in the outer leaflet of the IMM and the inner leaflet of the OMM—bind cyt *c* to form a peroxidase complex activated by the available H_2_O_2_. Deprotonated CL phosphate groups can electrostatically interact with eight positively charged lysine residues of cyt *c*, particularly Lys_72/73_ (74). The electrostatic binding is followed by strong hydrophobic interactions between PL acyl chains and non-polar regions of the protein (83). There are different views on the degree of the protein conformational changes induced by CL. While some of the data have been interpreted as the evidence for dramatic protein unfolding and denaturation (“molten globule”), recent multidimensional solid-state NMR results favors the model in which only minimal structural rearrangements take place whereby the hydrophilic milieu at the membrane interface stabilizes a native-like fold, but also leads to localized flexibility at the membrane-interacting protein face (84). One way or another, CL induced changes weaken and/or disrupt the coordination bond between heme-iron and Met_80_ (**Figure 2C**). Other amino acids can interact with the distal position of heme iron but they are not strong ligands and can be replaced by small molecules, including H_2_O_2_ and FA-OOH (73). Overall, the catalytic site of the cyt *c*/CL complex adopts peroxidase activity triggered by the available H_2_O_2_ or HOO-PUFA. If the iron in the cyt *c*/CL is in ferrous state, H_2_O_2_ oxidizes it into ferric form thus completing the conversion of cyt *c*/CL into a peroxidase. H_2_O_2_ leads to the formation of highly reactive oxoferryl porphyrin-π-cationic radical (compound I), which can oxidize various substrates (**Figure 3C**). In the case of pseudo-peroxidases, like cyt *c*/CL, compound I oxidizes protein amino acids to form a protein-immobilized radical (most likely tyrosyl) thus designating the emergence of compound II. Tyr_67_ is located in closest proximity to the heme group of cyt *c* and mediates oxidation of CL (**Figure 2C**) (85). The lower “pro-oxidant” capacity of compound II suggests that it is an unlikely candidate to further oxidize protein amino acids but it can readily abstract bis-allylic hydrogens from PUFA-CL (86). While other negatively charged lipids, like PIPs, PG, and PS, can also activate cyt *c* into a peroxidase, nevertheless the effectiveness of these alternative peroxidase complexes of cyt *c* with PLs is markedly lower than that of cyt *c*/CL complexes. Notably, the most abundant non-charged PLs of mitochondria, PC and PE, neither form peroxidase complexes with cyt *c* nor undergo peroxidation during apoptosis (16). Lipid hydroperoxides are orders of magnitude more effective in initiating CL peroxidation by cyt *c*/CL complexes than H_2_O_2_ (87). This indicates that accumulation of small amounts of HOO-CL may strongly stimulate the peroxidation process. Indeed, progressive acceleration of CL peroxidation in the presence of CL-OOH has been experimentally confirmed (74). The described role and specific features of CL peroxidation by cyt *c*/CL complexes may inform the mechanism-based design of small molecule anti-apoptotic regulators with therapeutic potential as described below (88, 89).

### 15LOX-Catalyzed Peroxidation of Phosphatidylethanolamines in Ferroptosis

The execution of ferroptosis includes the Fe-dependent production and accumulation of ox-PUFA-PL (27, 90). Theoretically, both an enzymatic mechanism as well as a random free radical reaction may be engaged in this process. As *sn2*-15-HpETE-PE has been identified as a selective and specific product eliciting pro-ferroptotic activity, it is reasonable to assume that an enzymatic mechanism should be, at least in part, enacted in ferroptosis. Among several possible redox-catalyzing Fe-proteins, 15LOX has been proposed as the likely candidate (34). Mammalian LOXes are a family of non-heme iron containing dioxygenases that effectively catalyze oxidation of one or more 1,4-cis,cis-pentadiene segments of free PUFA. A typical U-shaped PUFA binding channel is organized such that the oxidizable bis-allylic carbon is juxtaposed to Fe. The LOX nomenclature—5LOX, 8LOX, 12LOX, 15LOX—is based on the ETE carbon position that is oxidized by the enzyme’s Fe (91, 92). The highly organized catalytic site contains Fe^3+^ with five coordination bonds occupied by the protein’s amino acids and the sixth position interacting with the hydroxide (in the native enzyme) or water (in the intermediate) (**Figure 3D**). Fe^3+^-OH abstracts hydrogen from PUFA and yields a carbon-centered radical and Fe^2+^-OH_2_ (93). Thus formed lipid radical (L•) interacts with molecular oxygen (O_2_) delivered to the catalytic site through a special channel (94) which controls the production of the peroxyl radical at the catalytic site. The completion of the catalytic cycle is achieved *via* the hydrogen transfer from Fe^2+^-OH_2_ to the peroxyl radical resulting in the formation of the lipid hydroperoxide.

**Figure 4:**
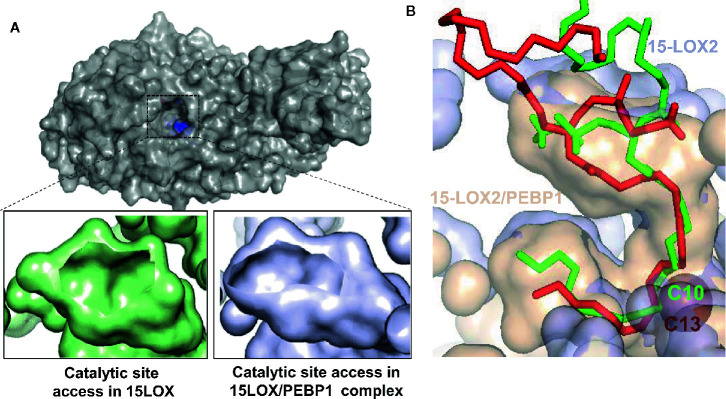
Allosteric modification of lipid binding in the 15LOX/PEBP1 complex. **(A)** Surface representation of 15LOX, viewed from top, showing the entrance to the catalytic site, the residues of which are highlighted in blue (top panel). The opening of the entrance site is reduced in the 15LOX/PEBP1 complex (bottom panel, right), compared to that in 15LOX alone (bottom panel, left). **(B)** The binding of sn1-18:0/sn2-20:4-PE onto 15LOX alone (red) and 15LOX/PEBP1 complex (green), showing the position of the nearest carbon at the catalytic iron. In 15LOX alone, this carbon is C10, leading to peroxidation at C13, where is in the complex it is C13, which leads to peroxidation at C15. These figures were adapted from (98).

Normally, 15LOX effectively oxidizes free ETE as a preferred substrate to generate 15-hydroperoxy-eicosatetraenoic acid (15HpETE) (71). Among the members of the LOX family, 15LOX is uniquely organized to also catalyze peroxidation of esterified PUFA, particularly membrane PLs (95). ETE-phosphatidylethanolamines (ETE-PE) represent one of the preferred substrates for 15LOX leading to the production of 15-HpETE-PE (96). The catalytic efficiency of 15LOX toward ETE-PE is relatively low. However, its formation has dramatic consequences as 15-HpETE-PE has been identified as a pro-ferroptotic signal (34). Paradoxically, a number of phenolic compounds and aromatic amines effectively prevent ferroptotic death but are poor 15LOX inhibitors (97). It has been hypothesized that 15LOX alone is not sufficient for the production of pro-ferroptotic death signals but there may be an additional factor modifying the enzymatic properties of 15LOX under ferroptotic conditions. Indeed, this factor has been identified as a scaffold protein, PE-binding protein-1 (PEBP1) (36). PEBP1 was shown to form a complex with 15LOX in which allosteric changes in 15LOX permit the entry and positioning of ETE-PE (**Figure 4**) in a way that the enzymatic activity toward specific oxidation of the ETE-residue increases two-fold (99). Participation of 15LOX/PEBP1 in the generation of pro-ferroptotic PEox death signals was demonstrated in a number of different types of cultured and primary cells as well as *in vivo* in airway epithelial cells in asthma, kidney epithelial cells in renal failure, cortical and hippocampal neurons in brain trauma (100), and in intestinal epithelial cells after total body irradiation (101). Given the demonstrated necro-inflammatory consequences of ferroptosis (102), the specific features of 15LOX/PEBP1 complexes offer an exciting opportunity for the design of ferroptosis-specific small molecule regulators with profound implications for human disease. It should be noted, however, that alternative enzymatic mechanisms of peroxidation of PUFA-PE may be involved in triggering ferroptosis. A recent study identified a NADPH-dependent oxidoreductase (possibly with a partner isoform of cytochrome P450), as an essential activator of pro-ferroptotic phospholipid peroxidation in several types of cancer cells with low levels of 15LOX expression (103). The sensitivity of cells to pro-ferroptotic stimulation was decreased after downregulation of the oxidoreductase but not of 15LOX. In contrast, in cells with high levels of 15LOX expression (e.g., after stimulation of human airways epithelial cells by inducers of Th2 responses), 15LOX KD caused a strong suppression of ferroptosis (36).

### Non-Enzymatic Lipid Peroxidation by “Loosely Bound” Fe-Complexes

Under extreme circumstances where the strict control of Fe is lost, Fe can display its redox activity mostly *via* participation in Fenton/Haber-Weiss reactions leading to the formation of ROS. The major biologically impactful event in these reactions is the reductive splitting of H_2_O_2_ by Fe^2+^ to yield highly reactive HO• radicals capable of initiating the oxidation process. H_2_O_2_ reacts poorly with most biological molecules due the high activation energy barrier that must be overcome (104). The rate constant of the reaction of H_2_O_2_ with free iron is low (< 10^2^ M^−1^s^−1^), however, Fe-ligation may accelerate the reaction by several orders of magnitude (up to 10^4^ M^−1^s^−1^). In the context of lipid peroxidation, HO• radicals avidly attack lipid molecules to produce carbon-centered lipid radicals (69) which, in the presence of O_2_, are converted into peroxyl radicals (LO_2_•). The latter are much less reactive toward lipids and the abstraction of a hydrogen atom from the oxidizable lipid molecules represents the “most difficult” initiating event. The easiest “victims” of RO_2_• are bis-allylic positions in PUFA—hence their number in PUFA is the major factor defining the oxidizability of lipids.

Random free radical chemical reactions are driven by the reactivity of the participating reagents—radicals and oxidation substrates and enzyme-imposed structural factors which do not limit the peroxidation process. Consequently, detection of selectivity of the peroxidation that deviates from this principle of oxidizability governed by the number of bis-allylic sites can be viewed as a strong argument against the participation of a free radical chemical reaction in the overall peroxidation process. Importantly, lipid peroxidation occurring during apoptosis and ferroptosis is highly specific not only with regards to classes of PLs—CL and PE. Indeed, linoleoyl (C18:2)-CLs represent the major substrates of pro-apoptotic peroxidation in mitochondria. Further, arachidonoyl (C20:4)- and adrenoyl (C22:4)-PE species, rather than more polyunsaturated docosapentaenoyl- and docosahexanoyl-PE species, are predominantly peroxidized in cells undergoing ferroptosis.

Another important difference between enzymatic and non-enzymatic LPO is that the latter is mostly driven by ferrous iron—in contrast to cyt c or 15LOX-dependent processes where ferric iron is the major catalytic species. In contrast, ferrous iron is markedly more effective in decomposing lipid hydroperoxides, the reaction leading to the production of secondary oxidatively truncated electrophilic products of LPO that can modify proteins and change their structure and functions. This is yet another controversy in understanding the leading role of Fe-dependent enzymatic *vs.* non-enzymatic reactions of LPO.

## Primary and Secondary Lipid Peroxidation Products

LPO—enzymatic or non-enzymatic—has a radical-mediated reaction in its nature. Radical intermediates are very short lived and cannot be directly detected by conventional high resolution analytical protocols such as LC-MS. The primary molecular products of LPO are hydroperoxides and they represent the first opportunity for the LC-MS based quantitative characterization. The analysis of lipid hydroperoxides has become a formidable task for several reasons. First, their chemical and metabolic instability and thermolabile nature most often results in the formation of secondary products, some of which are susceptible to degradation. Second, the sheer number of lipid signaling molecules is staggering which translates directly into a plethora of potential LPO products that, in many circumstances, occur at very low levels. Finally, while lipid hydroperoxides are the initial/primary products of lipid oxidation, secondary products such as aldehyde, ketone, hydroxy, and epoxide products add to the heterogeneity and complexity of the signaling language. Thus, this rich signaling language should include not only full-length peroxidation products, but also truncated PLs, cyclized PLs as well as fragments of oxidized fatty acyl chains resulting from secondary reactions of lipid hydroperoxides (34, 105).

**Figure 5:**
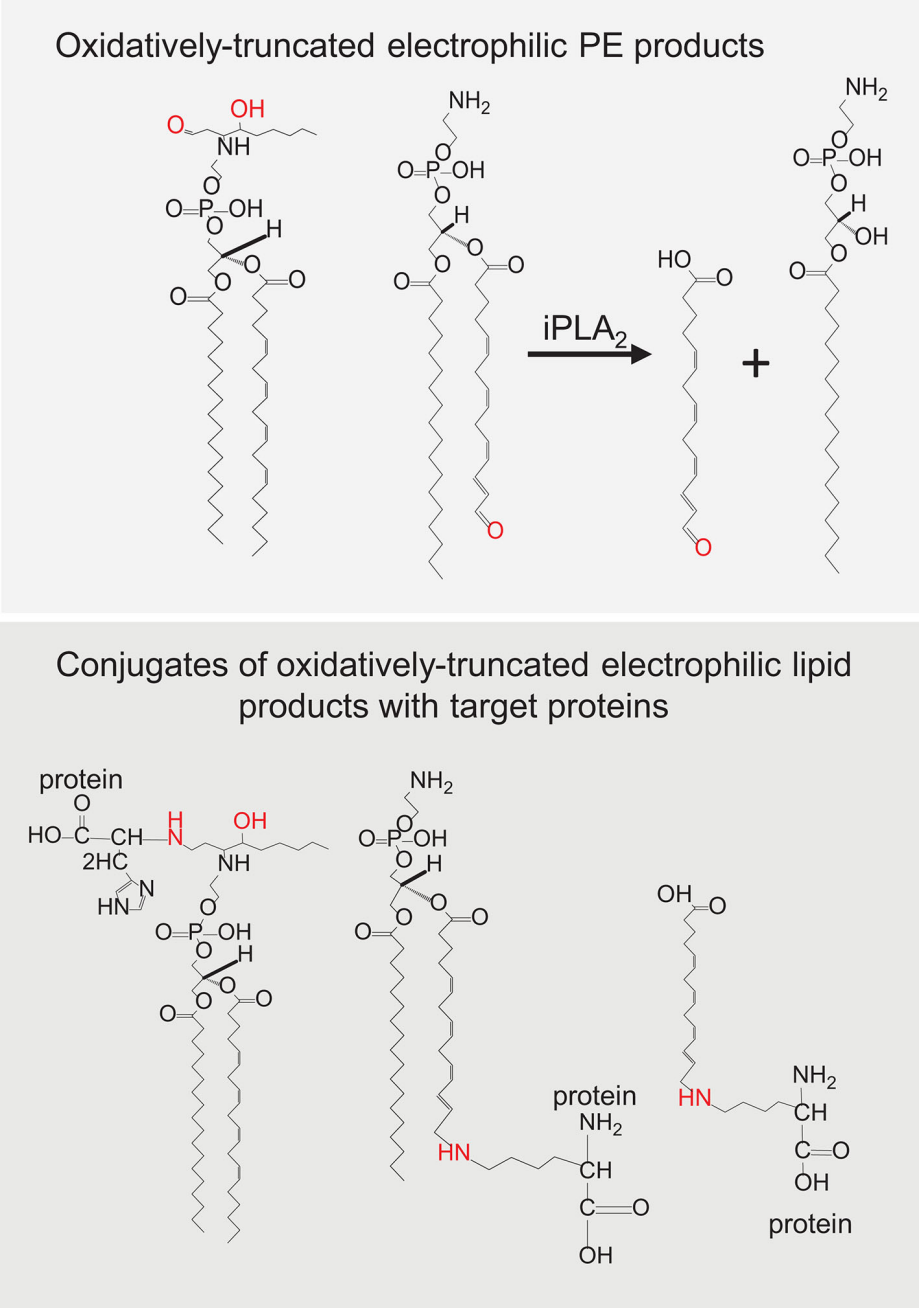
Oxidatively truncated electrophilic products formed from phosphatidylethanolamine (PE) and their conjugates with target proteins.

Lipid hydroperoxides are present in very low steady-state concentrations and are unstable due to their cleavage to yield, dependent on the reducing or oxidizing environment, new alkoxyl- or peroxyl- radicals, which readily decompose into secondary products (34). Indeed, many studies have focused on small reactive lipid fragments, such as malondialdehyde, 4-hydroxynonenal, etc. that can act as secondary downstream reactive products in a variety of cell death mechanisms and can covalently modify proteins [3]. However, this will depend on whether the reactive group (a reactive aldehyde for example) resides with the “leaving” short lipid fragment or remains with the truncated parent PL. Either of these two groups of reactive products can potentially react with nucleophilic amino acids, such as histidine, cysteine, and lysine that reside in cellular proteins. It has been well established that the process of PL peroxidation generates electrophilic species that are able to modify proteins and change their structure, activity and function (106). As PEs are preferentially peroxidized during ferroptotic death, it is likely that the truncated reactive parent PL is necessary for driving the downstream effects of ferroptosis (**Figure 5**). Indeed, small reactive lipid fragments can be formed from any polyunsaturated (phospho)lipid class, hence will not be specific for ferroptosis. By forming conjugates with a protein, lipidation by truncated reactive parent PLs will undoubtedly change the hydrophobic-hydrophilic balance, likely changing the distribution of the proteins into membranes (107–109) where they can form dreadful oligomeric pores.

Peroxidation reactions catalyzed by cyt *c*/CL complexes yield a highly diversified set of oxidized CL species with hydroperoxy-, hydroxy-, epoxy-, and oxo-functionalities (110). These CL peroxidation products are similar to the CLox signals detected in cells during execution of the intrinsic mitochondria-mediated apoptotic program triggered in cells by actinomycin D (16), staurosporine (17), and ionizing radiation (22, 111). Given that oxidizable PUFA-CLs are found exclusively in mitochondria, it is not surprising that oxidatively modified CL species, particularly mono-oxygenated C18:2-containing CLs, have been associated with the execution of the apoptotic death program (21). It should be noted, however, that a variety of CLox species, including hydroperoxy-, epoxy-, and oxo-CLs are detectable in cells and tissues *in vitro* and *in vivo*. For instance, CLox containing hydroxy-, epoxy-, oxo-, and hydroperoxy-functionalities have been detected in the small intestine of mice exposed to total body irradiation (110, 112, 113). Similarly, hydroxy- and hydroperoxy-CL species were detected in the brain of mice after traumatic injury (114). Notably, prevention of apoptosis by a mitochondrial electron acceptor, XJB-125, was associated with decreased levels of CLox in the peri-contusional zone of the traumatized brain.

**Figure 6:**
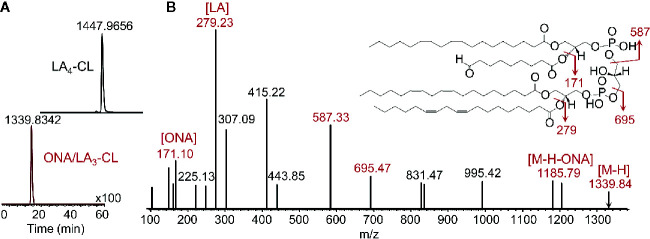
Enzymatic peroxidation of polyunsaturated CL by cyt c yields a variety of products (12, 110), including oxidatively truncated molecular species. Shown are LC-MS results identifying the production CL molecular species containing 9-oxo-nanoic acid. **(A)** Profiles of tetralinoleyl cardiolipin (LA_4_-CL, upper panel) with m/z 1,447.9656 and 9-oxo-nanoyl (ONA)/LA_3_-CL with m/z 1,339.8342 (lower panel); **(B)** MS^2^ fragmentation pattern and structural formulae (inset) of molecular ion with m/z 1,339.8342. MS^2^ analysis reveals the fragments with m/z 1,185.79, m/z 695.47, and 587.33 produced due to the loss of oxidatively truncated residue as well as to di-linoleoyl-glycerol phosphatidate and diacylglycerol phosphatidate containing linoleic acid (LA) and its oxidatively truncated residue. Further MS^2^ fragmentation of ion with m/z 587 yield ions with m/z 307 and m/z 415. The fragments corresponding to ONA (m/z 171) and LA (m/z 279) were detected as well. ONA, 9-oxo-nonanoic acid; LA, linoleic acid.

**Figure 7:**
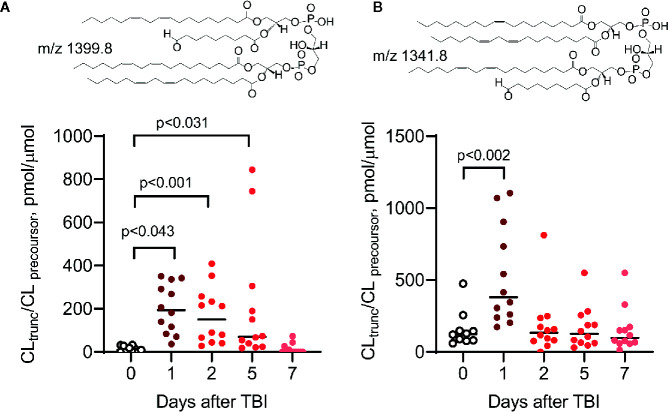
Peroxidized CLs, including oxidatively truncated species, are produced in the small intestine (ileum) of mice *in vivo* after total body irradiation (9.5 Gy) (110, 116). Two truncated CL species, ONA/LA_3_-CL **(A)** and OA/LA_2_/ONA-CL **(B)**, have been identified by MS^2^/MS^3^ fragmentation analysis (ONA, 9-oxo-nonanoic acid; LA, linoleic acid; OA, oleic acid). As previously described (110, 112, 113, 116), the levels of peroxidized CL (including oxidatively truncated CL species containing ONA) are elevated after irradiation. Insets: structural formulas of ONA/LA_3_-CL **(A)** and OA/LA/ONA-CL **(B)**.

It is possible that oxidatively truncated CLox species with high electrophilic potential can be formed in mitochondria. These products can readily attack nucleophilic sites in proteins and form protein adducts that will affect mitochondrial function and damage their integrity (115). Oxidatively truncated CL species can be formed in cyt *c*/CL catalyzed reactions *in vitro* (**Figure 6**) and were detected *in vivo* in the ileum of mice exposed to total body irradiation (**Figure 7**). While it is possible that truncated CLox products can modify a number of mitochondrial proteins, including those involved in the execution of mitochondria-dependent cell death programs (e.g., apoptosis), this work has not been accomplished and represents a goal for future investigations. The isolation and determination of the site of lipidation on proteins directly participating in the execution of cell death in apoptosis and ferroptosis is a formidable task. However, lipidated proteins, and more importantly lipidated peptides generated from protein digests, should impart a dominant hydrophobic characteristic whereby the peptides should be highly retained on reverse-phase solid supports. Adjusting and modifying gradients for extremely hydrophobic species and/or applying different chromatographic solid supports should aid in the separation and identification of lipidated species.

## Intracellular Localization of Lipid Peroxidation Centers

### Apoptosis

The execution of the intrinsic apoptotic program is initiated in mitochondria as it requires the oxidation of CLs (117). Formation of the peroxidase cyt *c*/CL complexes (16, 118) along with the H_2_O_2_-producing interruption in electron transport causes accumulation of CLox leading to the release of cyt *c* into the cytosol and apoptosome formation (75). In the cytosol, the released cyt *c* can bind an anionic phospholipid, phosphatidylserine (PS), located in the inner (cytosolic) leaflet of the plasma membrane. This complex also displays peroxidase activity that can trigger PS oxidation and accumulation of oxidized PS (PSox) (119, 120). As a membrane-disrupting agent, PSox can act as a “non-enzymatic scramblase” leading to the appearance of both PS and PSox molecules on the cell surface (120, 121). Both lipids serve as universal “eat-me” signals in efferocytosis, whereby PSox is more efficiently recognized by professional phagocytes (122–124). Thus, two separate cyt *c*-dependent oxidation mechanisms utilizing CL and PS are activated at different stages of apoptosis and generate signals with two distinctive functions. CLox is involved in the intracellular signaling at the initiation stage of apoptosis and leading to the release of cyt *c* from mitochondria, whereas PSox is an important part of inter-cellular communications regulating efferocytotic clearance of apoptotic cells by professional phagocytes (80, 125).

### Ferroptosis

Intracellular localization of LPO and participation of different organelles in the generation of ferroptotic death signal remains an important but still controversial issue (126, 127). Fluorescence-based measurements using a reagent selectively reacting with lipid hydroperoxides, Liperfluo, indicate that the endoplasmic reticulum (ER) is the major site of ferroptosis initiation (127). Both 15LOX and low molecular weight Fe-complexes are found in the ER compartment suggesting that both enzymatic initiation and non-enzymatic cleavage of HOO-PLs can take place in the ER (128, 129). The ER can also promote ferroptotic peroxidation indirectly *via* induction of autophagy driving the degradation of several important regulators of lipid peroxidation such as ferritin, lipid droplets and glutathione peroxidase 4 (GPX4) (130, 131). Given the likely engagement of mitochondria in the ferroptotic peroxidation, it is possible that mitochondria-associated ER membranes (MAMs) are the immediate locales where the LPO initiating events predominantly occur (132, 133).

Mitochondria are likely to be directly and/or indirectly involved in pro-ferroptotic LPO. There are several lines of evidence favoring the mitochondrial participation: i) GPX4 is localized in the intermembrane space of mitochondria, whereas 15LOX is associated with the mitochondrial membrane (94, 134–137), ii) a significant part (~40% of total phospholipids) of the major LPO substrate, PUFA-PE, is synthesized and present in this organelle (138), iii) they play a crucial role in cellular iron homeostasis, iv) they act as the major source of pro-oxidant ROS as well as a variety of molecular species of phospholipid hydroperoxides*, v)* they undergo dramatic morphological changes during ferroptosis (27). While strongly supportive, these characteristics make mitochondria a plausible but not penultimately proven universal participant of ferroptosis (139–141). It has been shown that cells incapable of generating mito-ROS due to depletion of mitochondrial DNA or with dramatically lowered levels of mitochondria (eliminated by mitophagy) did not demonstrate decreased sensitivity to ferroptosis (31). A highly effective mitochondria-targeted and hydrophobic radical scavenger, TPP-tagged MitoQ, was less effective than its non-targeted derivative (142). However, another mitochondria-targeted nitroxide, XJB-5-131, suppressed ferroptosis better than non-targeted nitroxides (143).

As an integral part of cell catabolism, lysosomes can, under some circumstances, indirectly participate in the ferroptotic program *via* autophagy e.g., by digesting Fe-containing proteins and releasing Fe used in pro-ferroptotic machinery (144, 145). However, it appears that lysosomal LPO is not critical for ferroptosis, as prevention of the accumulation of ferroptosis inhibitor ferrostatin-1 (Fer-1) in lysosomes, caused a stronger inhibition of ferroptosis (31). Evidently, the plasma membrane may be a target for the ferroptotic program rather than a part of its execution machinery and phospholipid peroxidation products may participate in the late destructive stages of the cell’s demise. In line with this, only minimal amounts of Fer-1 were detectable in the plasma membrane during execution of ferroptosis (146, 147).

### Non-Cell Autonomous Features of Lipid Signaling in Ferroptosis *vs.* Apoptosis

Preservation of plasma membrane integrity and formation of apoptotic bodies which are engulfed and removed by phagocytes are typical hallmarks of apoptosis. This process prevents spillover of cellular contents and makes apoptosis a non-inflammatory, cell-autonomous phenomenon. Opposite to apoptosis, ferroptotic cell death is “spread,” in a synchronized way, suggesting a direct cell-to-cell communication for the delivery of death signals (38, 148–150). The nature of potential death signals was revealed by LC-MS based redox lipidomics in a number of experiments, including those with the induction of ferroptosis by exogenous pLoxA, and included the oxidation of exogenous ETE-PE yielding 15-HpETE-PEs. Results of these experiments demonstrated the synchronous character of the spreading of cell death. The fluorescence of Liperfluo interacting with 15-HpETE-PEs demonstrated propagation of these products among the neighboring cells suggesting that they can serve as a death signal initiating ferroptosis (38). The non-cell autonomous nature of ferroptosis may be associated with intercellular communications stimulating immune and metabolic responses (38).

## Regulation of Lipid Peroxidation in Apoptosis and Ferroptosis and Its Specificity

The oxidation of CL during apoptosis occurs very early during the time when its molecular reactions leading to the initiation of apoptosis remain located in a restricted space inside of mitochondria whereby its inhibition provides an effective target for preventing apoptosis. This suggestion was confirmed by the experimental results demonstrating, that genetic depletion of CL-synthase as well as deficiency of cyt c or mutation of its Tyr_67_ residue result in an increased resistance of cells to apoptosis (20, 151–153). Several low molecular weight compounds preventing CL oxidation by inhibiting of peroxidase activity of cyt c have been designed and tested, including mitochondria-targeted electron scavengers and stable nitroxides radicals (116, 154–156).

Regulation of LPO which play crucial roles in the development of ferroptosis is a key approach in preventing ferroptotic cell death. One of the most important defenders is glutathione peroxidase 4 (Gpx4), converting toxic PL hydroperoxides to non-toxic alcohols by using GSH as the reducing *substrate* (142). Direct inactivation of GPX4 by small molecular weight compounds interacting with selenocysteine such as RSL3, ML162, ML210, FINO_2_ as well as indirect inhibition by deprivation of GSH were effective in inducing ferroptosis (28, 126, 157) (**Table 1**). One of the prerequisites for ferroptosis is the presence of ETE-PE, the substrate for the production of the death signal, HpETE-PE. Enzymes participating in the synthesis of ETE-PE such as ACSL4 responsible for the formation of CoA-derivatives of ETE and LPCAT3 catalyzing the esterification of CoA-ETE into lyso-PE, act upstream of GPX4 and are important ferroptosis regulators. Experimental data demonstrated a direct correlation between their expression and sensitivity to ferroptosis (34–36). GPX4 and ACSL4 double-KO cells have the ability to overcome the deadly effect of GPX4 deficiency and do not undergo ferroptosis (35).

Some cells contain additional enzymatic mechanisms such as inducible nitric oxide synthase (iNOS)/NO• which can interact with 15LOX and/or lipid radicals and neutralize them (37). The anti-ferroptotic effect of NO• involves the inhibition of 15-LOX-dependent oxidation of ETE-PE and neutralization of HpETE-PE as well as secondary lipid radicals formed during cleavage and oxidative truncation of this molecule, thus preventing their toxic effects. Increased expression of iNOS/NO• in M1 macrophages/microglia or addition of NO• donors to the M2 macrophages not expressing iNOS lead to their high resistance to ferroptosis. Due to its ability to neutralize the formation of HpETE-PE, iNOS can inhibit ferroptotic cell death acting upstream of GPX4.

Nrf2/NFE2L2 transcription factor is activated as a feedback loop to protect cells from LPO generated during ferroptosis and it is one of the key players in the protection of cells from ferroptosis. After dissociation from its complex with its negative regulator Keap1, Nrf2 translocates to the nucleus where it activates the transcription of target genes (158, 159). NRF2 inhibits ferroptosis through the regulation of hundreds of genes, including genes participating in the regulation of glutathione, GPX4 expression, iron, mitochondrial function and lipid metabolism (159, 160).

### Unique Role of Thiols

Reduced glutathione (GSH), the most prevalent non-protein thiol and the major intracellular antioxidant, plays an important role in maintaining a tight control over the redox status and cellular defense against LPO. However, the specific mechanisms of action of GSH in apoptosis and ferroptosis are different in spite of the fact that GSH depletion is a common feature of both extrinsic and intrinsic apoptosis (161). GSH depletion creates redox instability promoting the activation of signaling pathways leading to the initiation of apoptosis. One of the possible mechanisms activated by GSH depletion involves the deterioration of mitochondrial function. It appears that GSH deficiency promotes pro-apoptotic effects of other inducers; by itself GSH depletion is not sufficient to initiate apoptosis (162).

In ferroptosis, GSH is required as a substrate for proper functioning of GPX4. A decline in the GSH contents leads to the accumulation of pro-ferroptotic PUFA-PLox. The cystine/glutamate antiporter (system xc^−^) represents the main route for extracellular transport of cystine serving as an essential precursor for the synthesis of GSH. The xc^−^/GSH/GPX4 axis is the crucial controlling mechanism of ferroptosis and its inhibitors (e.g., erastin, imidazole ketone erastin, sulfasalazine, etc.) are classified as “class 1” ferroptosis-inducing compounds to distinguish them from RSL3 and other direct inhibitors of GPX4 classified as “class 2” (163).

### Thiol-Independent Regulation of Lipid Signaling

A recently discovered protein FSP-1—formerly known as mitochondrial flavoprotein AIFM2—was found to inhibit ferroptosis in a GPX4/GSH-*independent* way. Accordingly, FSP1 knockout cells display a higher sensitivity to ferroptosis inducers whereas overexpression of FSP1 correlates with a higher resistance to ferroptosis in multiple tumor cell lines. The mechanisms of FSP-1 anti-ferroptotic effects involve its ability to serve as an oxidoreductase catalyzing the NAD(P)H-dependent reduction of CoQ to ubiquinol (43, 44).

Similar to vitamin E, a lipophilic radical-trapping antioxidant CoQ10 is a very effective inhibitor of ferroptosis both *in vitro* and *in vivo* (164, 165). The redox biochemistry of CoQ and vitamin E are intertwined: the more powerful antioxidant, vitamin E (tocopherol), neutralizes lipid radicals by donating an electron and can be regenerated back to its reduced form by CoQ-OH (166–169). This recycling of vitamin E is maintained by FSP1 that maintains control of the reduced CoQ-OH. In addition to FSP-1, cells contain several oxidoreductases capable of reducing CoQ and vitamin E, hence be involved in the GSH/GPX4 independent inhibition of ferroptosis. Additional work in this area will elucidate the role of several oxidoreductases such as NAD(P)H: (quinone acceptor) oxidoreductase 1 (NQO1) (170), cytochrome *b*5 reductase (CyB5R), in CoQ/vitamin E anti-ferroptotic regulations (171).

## Mechanism-Based Regulators of Enzymatic Phospholipid Peroxidation as Specific Anti-Apoptotic and Anti-Ferroptotic Agents

Contemporary understanding of the role, contribution and mechanisms of PL peroxidation as drivers of apoptotic and ferroptotic death programs, leads to the design of new classes of specific small molecule inhibitors with a potential for their application as therapeutic agents. To emphasize the importance of the structural and functional organization of the enzymatic complexes directly involved in the production of PL death signals, we will re-iterate their relevant and most important features in this section.

### Anti-Apoptotic Regulators

Oxidation of mitochondrial CL by the cyt *c*/CL peroxidase complex is an early and critical step in apoptosis signaling. CL oxidation facilitates the release of cyt *c* from mitochondria to the cytosol and participates in apoptosome formation. CL depletion disrupts the function of various IMM enzymatic complexes (80, 172). Regulating enzymatic CL oxidation is a promising LPO-focused anti-apoptotic therapeutic strategy. CL is mitochondria-specific and asymmetrically localized, being overwhelmingly localized and enriched (≤25% of total PL) at the IMM inner leaflet. To interact with intermembrane cyt *c*, CL is first flipped to the IMM outer leaflet. The mechanisms of the overall process leading to CL externalization remain enigmatic. Several candidate proteins contribute to loss of CL asymmetry following injury—such as PL scramblase-3 (PLS3) that facilitates transposition of CL from the inner-to-outer IMM leaflets and other transporters implicated in IMM-to-OMM CL transport [e.g., mitochondrial nucleoside diphosphate kinase (NDPKD), adenine nucleotide translocator (ANT), uncoupling proteins (uCP), creatine phosphokinase (CPK), and truncated-BH3 interacting domain death agonist (t-Bid)] (173, 174).

Interaction between CL and cyt *c* is mediated by electrostatic forces between positively charged lysines in cyt *c* and CL’s two negative phosphate groups (74, 175). Binding of CL induces: **1**) distortion of cyt *c*’s heme-associated Trp_59_ and reduction in cyt *c*’s hydrophobic core volume; **2**) disruption of cyt *c*’s Met_80_-Fe bond, lead to a mixture of penta-coordinated and His_33_/His_26_ hexa-coordinated heme (176); **3**) a decreased Fe(II)/Fe(III) couple redox potential (~400 mV more negative than native cyt *c*) abolishing its electron shuttling activity while gaining peroxidase activity (177, 178); and **4**) opening of cyt *c*’s heme crevice enabling substrate access to the newly formed peroxidase active site (153, 179). The newly formed cyt *c*/CL peroxidase substrate specificity leans toward organic peroxides, like CLox. However, given the complex’s proximity to mitochondrial H_2_O_2_ sources (ETC complexes and TCA dehydrogenase) and electrostatic affinity for unoxidized CL, the primary substrate of the cyt *c*/CL complex is CLox (180, 181). Liberation of CL’s oxidized acyl chains through the action of phospholipases (e.g., iPLA_2_γ) yields a suite of immune activating signaling molecules—such as hydroxy-octadecadienoic acids (HODEs), hydroperoxyoctadecadienoic acids (HpODEs), and hydroxy-eicosatetraenoic acids (HETEs) (182, 183).

**Figure 8:**
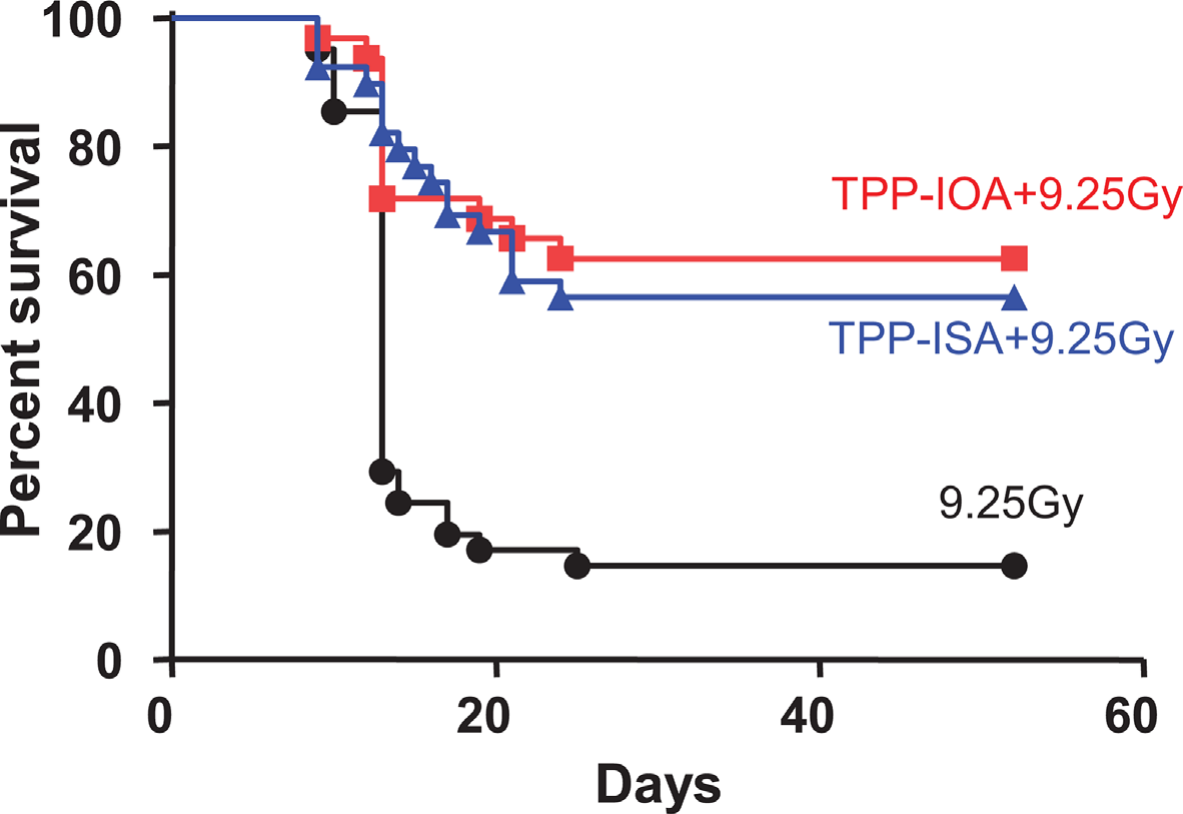
Radiation protection and mitigation by TPP-IOA and TPP-ISA. C57BL/6NTac female mice were exposed to total body irradiation to a dose of 9.25 Gy using a cesium source (*n* = 31–35 mice per group). The mice were irradiated and injected i.p. with TPP-IOA or TPP-ISA (5 mg per kg body weight in 100 μl of water containing 25% ethanol) 10 min after irradiation. *P* < 0.0001 (a two-sided log-rank test)—TPP-IOA or TPP-ISA injected and exposed to total body irradiation mice *vs.* mice exposed to total body irradiation only. These figure was adapted from (116).

Small molecule therapies targeting CL peroxidation hold promise as a specific anti-apoptotic interventions. However, the administration of non-targeted global antioxidants (e.g., vitamin E, CoQ10, or nitroxyl radicals) proved ineffective at improving survival and CLox-associated apoptosis in acute injury models. Poor localization of the therapeutic compounds to the pathogenic target sites, low activity with the target substrate, and/or deleterious off-target effects on ROS signaling may have led to this failure (184). These limitations may all be overcome, to varying degrees, by specifically targeting and enriching antioxidant and electron scavengers, like nitroxide, to the IMM. XJB-5-131 and JP4-039 are comprised of a hemigramicidin (HS) peptide conjugated to TEMPO (114). These compounds attenuate lesion volume and improve behavioral deficits following experimental brain injury. Mechanistically, XJB-5-131 serves as an electron scavengers, preventing $O_{2}^{\cdot -}$ and H_2_O_2_ formation and limiting fuel for the cyt *c*/CL peroxidase. Alternatively, mitochondrial-targeted imidazole-conjugated fatty acids (TPP-ISA, TPP-IOA) coordinate the hepta-coordinated form of the cyt *c*/CL complex, impeding CL access and oxidation (**Figure 8**) (80). Therapies may also promote CL lipidome remodeling into a less oxidizable, mono-unsaturated form using mitochondria targeted oleic or stearic acid derivatives (185). While perhaps less viable for application in acute injury, this remodeling approach has shown potential in chronic neurodegenerative disease models (114). Development of new mitochondria-targeted molecules that suppress CL peroxidation, as well as the optimization of the pharmacodynamic/pharmacokinetic properties of existing ones may lead to promising ant-apoptotic therapeutic modalities.

### Anti-Ferroptotic Regulators

Most anti-ferroptosis strategies target LPO in one way or another—whether they promote cellular lipidome remodeling into a less oxidizable form, inhibit LOXs, or serve as lipophilic radical trapping agents. As indicated above, many of the ferroptotic inhibitors exhibit both LOX inhibition and radical trapping capacity, although to varying degrees (186, 187). The lack of appropriate analytical tools hindered the understanding of the precise mechanism of these molecules. With regards to the LOX inhibitors, their anti-ferroptotic effectiveness is often higher than the LOX inhibitory activity (97). Notably, the EC_50_ values for LOX inhibition have been calculated exclusively based on the suppression of free HpETE production by LOX alone. The rate of the HpETE production by 15LOX is many-fold higher than that of HpETE-PE. Because the latter, but not free HpETE, act as pro-ferroptotic signals, the meaning of these estimates in the context of ferroptosis is dubious (34). Assuming that the 15LOX/PEBP1 complex is the generator of pro-ferroptotic HpETE-PE signals (36, 188), assessments of small molecule radical scavengers have to be performed in this system. A recent study conducted with Fer-1, known to act as good radical scavenger, demonstrated that the EC_50_ value for Fer-1 in inhibiting the HpETE-PE production by the 15LOX/PEBP1 complex is considerably lower than its effective cytoprotective concentration in cells triggered to undergo ferroptosis (98). Moreover, the Fer-1-driven ferroptosis suppression was mainly realized through its enzymatic inhibitory capacity (98). It should be noted that the radical trapping reagents could have off-target effects that may decrease the (phospho)lipid peroxide load of the cellular system. These effects may be helpful to mitigate diseases involving multimodal mechanisms of cell death as exemplified by traumatic brain injury and inflammatory disease triggered by total body gamma-irradiation (36, 46, 189). At the same time, highly specific inhibitors could be beneficial for diseases in which ferroptosis is the predominant pathogenic mechanism.

## Concluding Remarks

PLs are the major building blocks of the membrane bilayer and their structural role is indispensable and fundamental to compartmentalization and interfacing of thousands of processes in cells and their organelles. In this capacity, macroscopic characteristics of the PL assembles such as fluidity, flexibility, lateral and trans-membrane diffusion are essential for the function of membranes as interactive barriers. Interconnected with this is the signaling by membrane phospholipids that is associated with their post-synthetic modifications. Most commonly this occurs *via* oxidative transformations of polyunsaturated PL resulting in the production of a huge variety of new molecular species of lipids, integrated into a new concept of the *epilipidome*. The epilipidome includes not only oxidatively modified lipids but also a huge variety of their adducts with proteins thus merging with the epi-proteome, post-translationally modified proteins. Signaling by individual lipids and lipoprotein adducts comprising the epilipidome/epiproteome is broadly employed in almost each of the myriads of life-defining activities of cells and cell communities. This type of signaling is also essential for coordination of regulated cell death programs. Although these programs may be viewed as suicidal, their strict control and harmonized execution defines the borderline between health and disease. Therefore, deciphering the signaling phospholipid language of these programs is one of the central areas of research not only in cell biology but also in many fields of biomedicine.

While lipid peroxidation has been attributed to essentially all known death programs, its specific mechanisms and role have been clearly defined for only two of them—apoptosis and ferroptosis. Formation of free radical intermediates in the course of LPO occurs both during regulated enzymatic and random non-enzymatic processes. As a result of this, a broad spectrum of agents of different classes with high hydrogen/electron-donating capacities, particularly phenolic compounds and aromatic amines, may be effective in blocking the LPO component of death programs. This has created an optimistic view that many of these compounds may lead to “anti-suicidal” cell-based therapies. The indiscriminative nature of this strategy—likely affecting multiple vital biochemical reactions proceeding *via* free radical intermediates—may be associated with low effectiveness and serious side effects. Free radical scavengers/sacrificial antioxidants/chain-breaking antioxidants—have been designed and developed and ultimately tested in numerous clinical trials in more than three dozens of diseases. Disappointingly, the results of these were uniformly negative. This strategic mistake in searches for a “magic antioxidant bullet” should be avoided in the design of new anti-apoptotic and anti-ferroptotic therapeutic agents—the lesson has to be learnt. Future generations of small molecule regulators of *regulated* cell death program therapies must consider their selective and specific mechanisms, hence, to be precise and highly discriminative. In line with this, distinctive inhibitors/regulators have to be developed for controlling individual cell death programs—as has been discussed above.

## Author Contributions

VEK conceived the idea and wrote the manuscript. YYT, IIV, IHS, and VAT prepared the figures and wrote part of the manuscript. AAK, AAA, TSA FBC, AL, MWE, JSG, DHB, RKM, AKS, HB, and AAS wrote part of the manuscript. All authors contributed to the article and approved the submitted version.

## Funding

This work was supported by NIH (HL114453, AI156924, AI156923, CA165065, CA243142, AI145406, NS076511, and NS061817) and grant No. 075-15-2020-926 World-Class Research Center (Ministry of Science and Higher Education of the Russian Federation). This project has been funded in whole or in part with federal funds from the National Cancer Institute, National Institutes of Health, under Contract No. HHSN261200800001E.

## Disclaimer

The content of this publication does not necessarily reflect the views or policies of the Department of Health and Human Services, nor does mention of trade names, commercial products, or organizations imply endorsement by the U.S. Government.

The findings and conclusions in this report are those of the authors and do not necessarily represent the official position of the National Institute for Occupational Safety and Health, Centers for Disease Control and Prevention.

## Conflict of Interest

The authors declare that the research was conducted in the absence of any commercial or financial relationships that could be construed as a potential conflict of interest.

## References

[1] Kunkel TA Bebenek K. DNA replication fidelity. Annu Rev Biochem (2000) 69:497–529. 10.1146/annurev.biochem.69.1.497 10966467

[2] Nguyen LK Kolch W Kholodenko BN. When ubiquitination meets phosphorylation: a systems biology perspective of EGFR/MAPK signalling. Cell Commun Signal (2013) 11:52. 10.1186/1478-811X-11-52 23902637 PMC3734146

[3] Linder ME Deschenes RJ. Palmitoylation: policing protein stability and traffic. Nat Rev Mol Cell Biol (2007) 8:74–84. 10.1038/nrm2084 17183362

[4] Ponomarenko EA Poverennaya EV Ilgisonis EV Pyatnitskiy MA Kopylov AT Zgoda VG. The Size of the Human Proteome: The Width and Depth. Int J Anal Chem (2016) 2016:7436849. 10.1155/2016/7436849 27298622 PMC4889822

[5] Aebersold R Agar JN Amster IJ Baker MS Bertozzi CR Boja ES. How many human proteoforms are there? Nat Chem Biol (2018) 14:206–14. 10.1038/nchembio.2576 PMC5837046 29443976

[6] Kagan VE Tyurina YY Sun WY Vlasova II Dar H Tyurin VA. Redox phospholipidomics of enzymatically generated oxygenated phospholipids as specific signals of programmed cell death. Free Radic Biol Med (2020) 147:231–41. 10.1016/j.freeradbiomed.2019.12.028 PMC7037592 31883467

[7] Wiernicki B Dubois H Tyurina YY Hassannia B Bayir H Kagan VE. Excessive phospholipid peroxidation distinguishes ferroptosis from other cell death modes including pyroptosis. Cell Death Dis (2020) 11:922. 10.1038/s41419-020-03118-0 33110056 PMC7591475

[8] Ni Z Goracci L Cruciani G Fedorova M. Computational solutions in redox lipidomics - Current strategies and future perspectives. Free Radic Biol Med (2019) 144:110–23. 10.1016/j.freeradbiomed.2019.04.027 31035005

[9] Shaikh SR Edidin M. Polyunsaturated fatty acids and membrane organization: elucidating mechanisms to balance immunotherapy and susceptibility to infection. Chem Phys Lipids (2008) 153:24–33. 10.1016/j.chemphyslip.2008.02.008 18346461 PMC2442228

[10] Kagan VE. Lipid peroxidation in biomembranes. Boca Raton: CRC Press (1988).

[11] Niki E. Biomarkers of lipid peroxidation in clinical material. Biochim Biophys Acta (2014) 1840:809–17. 10.1016/j.bbagen.2013.03.020 23541987

[12] Tyurina YY Tyurin VA Anthonymuthu T Amoscato AA Sparvero LJ Nesterova AM. Redox lipidomics technology: Looking for a needle in a haystack. Chem Phys Lipids (2019) 221:93–107. 10.1016/j.chemphyslip.2019.03.012 30928338 PMC6714565

[13] Cruciani G Domingues P Fedorova M Galli F Spickett CM. Redox lipidomics and adductomics - Advanced analytical strategies to study oxidized lipids and lipid-protein adducts. Free Radic Biol Med (2019) 144:1–5. 10.1016/j.freeradbiomed.2019.07.027 31369839

[14] Fedorova M Bollineni RC Hoffmann R. Protein carbonylation as a major hallmark of oxidative damage: update of analytical strategies. Mass Spectrom Rev (2014) 33:79–97. 10.1002/mas.21381 23832618

[15] Tang D Kang R Berghe TV Vandenabeele P Kroemer G. The molecular machinery of regulated cell death. Cell Res (2019) 29:347–64. 10.1038/s41422-019-0164-5 PMC6796845 30948788

[16] Kagan VE Tyurin VA Jiang J Tyurina YY Ritov VB Amoscato AA. Cytochrome c acts as a cardiolipin oxygenase required for release of proapoptotic factors. Nat Chem Biol (2005) 1:223–32. 10.1038/nchembio727 16408039

[17] Tyurin VA Tyurina YY Feng W Mnuskin A Jiang J Tang M. Mass-spectrometric characterization of phospholipids and their primary peroxidation products in rat cortical neurons during staurosporine-induced apoptosis. J Neurochem (2008) 107:1614–33. 10.1111/j.1471-4159.2008.05728.x PMC2760311 19014376

[18] Tyurina YY Tyurin VA Kaynar AM Kapralova VI Wasserloos K Li J. Oxidative lipidomics of hyperoxic acute lung injury: mass spectrometric characterization of cardiolipin and phosphatidylserine peroxidation. Am J Physiol Lung Cell Mol Physiol (2010) 299:L73–85. 10.1152/ajplung.00035.2010 PMC2904094 20418384

[19] Tyurina YY Winnica DE Kapralova VI Kapralov AA Tyurin VA Kagan VE. LC/MS characterization of rotenone induced cardiolipin oxidation in human lymphocytes: implications for mitochondrial dysfunction associated with Parkinson’s disease. Mol Nutr Food Res (2013) 57:1410–22. 10.1002/mnfr.201200801 PMC3810210 23650208

[20] Huang Z Jiang J Tyurin VA Zhao Q Mnuskin A Ren J. Cardiolipin deficiency leads to decreased cardiolipin peroxidation and increased resistance of cells to apoptosis. Free Radic Biol Med (2008) 44:1935–44. 10.1016/j.freeradbiomed.2008.02.016 PMC2692820 18375209

[21] Mao G Qu F St Croix CM Tyurina YY Planas-Iglesias J Jiang J. Mitochondrial Redox Opto-Lipidomics Reveals Mono-Oxygenated Cardiolipins as Pro-Apoptotic Death Signals. ACS Chem Biol (2016) 11:530–40. 10.1021/acschembio.5b00737 PMC5741079 26697918

[22] Belikova NA Jiang J Tyurina YY Zhao Q Epperly MW Greenberger J. Cardiolipin-specific peroxidase reactions of cytochrome C in mitochondria during irradiation-induced apoptosis. Int J Radiat Oncol Biol Phys (2007) 69:176–86. 10.1016/j.ijrobp.2007.03.043 17707271

[23] Bayir H Tyurin VA Tyurina YY Viner R Ritov V Amoscato AA. Selective early cardiolipin peroxidation after traumatic brain injury: an oxidative lipidomics analysis. Ann Neurol (2007) 62:154–69. 10.1002/ana.21168 17685468

[24] Ji J Tyurina YY Tang M Feng W Stolz DB Clark RS. Mitochondrial injury after mechanical stretch of cortical neurons in vitro: biomarkers of apoptosis and selective peroxidation of anionic phospholipids. J Neurotrauma (2012) 29:776–88. 10.1089/neu.2010.1602 PMC3303096 21895519

[25] Jiang J Kini V Belikova N Serinkan BF Borisenko GG Tyurina YY. Cytochrome c release is required for phosphatidylserine peroxidation during Fas-triggered apoptosis in lung epithelial A549 cells. Lipids (2004) 39:1133–42. 10.1007/s11745-004-1340-1 15726829

[26] Serinkan BF Tyurina YY Babu H Djukic M Quinn PJ Schroit A. Vitamin E inhibits anti-Fas-induced phosphatidylserine oxidation but does not affect its externalization during apoptosis in Jurkat T cells and their phagocytosis by J774A.1 macrophages. Antioxid Redox Signal (2004) 6:227–36. 10.1089/152308604322899297 15025924

[27] Dixon SJ Lemberg KM Lamprecht MR Skouta R Zaitsev EM Gleason CE. Ferroptosis: an iron-dependent form of nonapoptotic cell death. Cell (2012) 149:1060–72. 10.1016/j.cell.2012.03.042 PMC3367386 22632970

[28] Yang WS Kim KJ Gaschler MM Patel M Shchepinov MS Stockwell BR. Peroxidation of polyunsaturated fatty acids by lipoxygenases drives ferroptosis. Proc Natl Acad Sci USA (2016) 113:E4966–4975. 10.1073/pnas.1603244113 PMC5003261 27506793

[29] Larraufie MH Yang WS Jian E Thomas AG Slusher BS Stockwell BR. Incorporation of metabolically stable ketones into a small molecule probe to increase potency and water solubility. Bioorg Med Chem Lett (2015) 25:4787–92. 10.1016/j.bmcl.2015.07.018 PMC4653046 26231156

[30] Louandre C Ezzoukhry Z Godin C Barbare JC Maziere JC Chauffert B. Iron-dependent cell death of hepatocellular carcinoma cells exposed to sorafenib. Int J Cancer (2013) 133:1732–42. 10.1002/ijc.28159 23505071

[31] Gaschler MM Andia AA Liu H Csuka JM Hurlocker B Vaiana CA. Determination of the Subcellular Localization and Mechanism of Action of Ferrostatins in Suppressing Ferroptosis. ACS Chem Biol (2018) 13:1013–20. 10.1021/acschembio.8b00199 PMC5960802 29512999

[32] Yang WS SriRamaratnam R Welsch ME Shimada K Skouta R Viswanathan VS. Regulation of ferroptotic cancer cell death by GPX4. Cell (2014) 156:317–31. 10.1016/j.cell.2013.12.010 PMC4076414 24439385

[33] Basit F van Oppen LM Schockel L Bossenbroek HM van Emst-de Vries SE Hermeling JC. Mitochondrial complex I inhibition triggers a mitophagy-dependent ROS increase leading to necroptosis and ferroptosis in melanoma cells. Cell Death Dis (2017) 8:e2716. 10.1038/cddis.2017.133 28358377 PMC5386536

[34] Kagan VE Mao G Qu F Angeli JP Doll S Croix CS. Oxidized arachidonic and adrenic PEs navigate cells to ferroptosis. Nat Chem Biol (2017) 13:81–90. 10.1038/nchembio.2238 27842066 PMC5506843

[35] Doll S Proneth B Tyurina YY Panzilius E Kobayashi S Ingold I. ACSL4 dictates ferroptosis sensitivity by shaping cellular lipid composition. Nat Chem Biol (2017) 13:91–8. 10.1038/nchembio.2239 PMC5610546 27842070

[36] Wenzel SE Tyurina YY Zhao J St Croix CM Dar HH Mao G. PEBP1 Wardens Ferroptosis by Enabling Lipoxygenase Generation of Lipid Death Signals. Cell (2017) 171:628–641 e626. 10.1016/j.cell.2017.09.044 29053969 PMC5683852

[37] Kapralov AA Yang Q Dar HH Tyurina YY Anthonymuthu TS Kim R. Redox lipid reprogramming commands susceptibility of macrophages and microglia to ferroptotic death. Nat Chem Biol (2020) 16:278–90. 10.1038/s41589-019-0462-8 PMC7233108 32080625

[38] Dar HH Tyurina YY Mikulska-Ruminska K Shrivastava I Ting HC Tyurin VA. Pseudomonas aeruginosa utilizes host polyunsaturated phosphatidylethanolamines to trigger theft-ferroptosis in bronchial epithelium. J Clin Invest (2018) 128:4639–53. 10.1172/JCI99490 PMC6159971 30198910

[39] Hassannia B Wiernicki B Ingold I Qu F Van Herck S Tyurina YY. Nano-targeted induction of dual ferroptotic mechanisms eradicates high-risk neuroblastoma. J Clin Invest (2018) 128:3341–55. 10.1172/JCI99032 PMC6063467 29939160

[40] Yoshida M Minagawa S Araya J Sakamoto T Hara H Tsubouchi K. Involvement of cigarette smoke-induced epithelial cell ferroptosis in COPD pathogenesis. Nat Commun (2019) 10:3145. 10.1038/s41467-019-10991-7 31316058 PMC6637122

[41] Eling N Reuter L Hazin J Hamacher-Brady A Brady NR. Identification of artesunate as a specific activator of ferroptosis in pancreatic cancer cells. Oncoscience (2015) 2:517–32. 10.18632/oncoscience.160 PMC4468338 26097885

[42] Lisewski AM Quiros JP Ng CL Adikesavan AK Miura K Putluri N. Supergenomic network compression and the discovery of EXP1 as a glutathione transferase inhibited by artesunate. Cell (2014) 158:916–28. 10.1016/j.cell.2014.07.011 PMC4167585 25126794

[43] Bersuker K Hendricks JM Li Z Magtanong L Ford B Tang PH. The CoQ oxidoreductase FSP1 acts parallel to GPX4 to inhibit ferroptosis. Nature (2019) 575:688–92. 10.1038/s41586-019-1705-2 PMC6883167 31634900

[44] Doll S Freitas FP Shah R Aldrovandi M da Silva MC Ingold I. FSP1 is a glutathione-independent ferroptosis suppressor. Nature (2019) 575:693–8. 10.1038/s41586-019-1707-0 31634899

[45] Matsushita M Freigang S Schneider C Conrad M Bornkamm GW Kopf M. T cell lipid peroxidation induces ferroptosis and prevents immunity to infection. J Exp Med (2015) 212:555–68. 10.1084/jem.20140857 PMC4387287 25824823

[46] Kenny EM Fidan E Yang Q Anthonymuthu TS New LA Meyer EA. Ferroptosis Contributes to Neuronal Death and Functional Outcome After Traumatic Brain Injury. Crit Care Med (2019) 47:410–8. 10.1097/CCM.0000000000003555 PMC6449247 30531185

[47] Li W Feng G Gauthier JM Lokshina I Higashikubo R Evans S. Ferroptotic cell death and TLR4/Trif signaling initiate neutrophil recruitment after heart transplantation. J Clin Invest (2019) 129:2293–304. 10.1172/JCI126428 PMC6546457 30830879

[48] Laird MD Wakade C Alleyne CH Jr Dhandapani KM. Hemin-induced necroptosis involves glutathione depletion in mouse astrocytes. Free Radic Biol Med (2008) 45:1103–14. 10.1016/j.freeradbiomed.2008.07.003 18706498

[49] Chang YJ Hsu SL Liu YT Lin YH Lin MH Huang SJ. Gallic acid induces necroptosis via TNF-alpha signaling pathway in activated hepatic stellate cells. PloS One (2015) 10:e0120713. 10.1371/journal.pone.0120713 25816210 PMC4376672

[50] Ghardashi Afousi A Gaeini A Rakhshan K Naderi N Darbandi Azar A Aboutaleb N. Targeting necroptotic cell death pathway by high-intensity interval training (HIIT) decreases development of post-ischemic adverse remodelling after myocardial ischemia / reperfusion injury. J Cell Commun Signal (2019) 13:255–67. 10.1007/s12079-018-0481-3 PMC6498245 30073629

[51] Canli O Alankus YB Grootjans S Vegi N Hultner L Hoppe PS. Glutathione peroxidase 4 prevents necroptosis in mouse erythroid precursors. Blood (2016) 127:139–48. 10.1182/blood-2015-06-654194 PMC4705604 26463424

[52] Kang R Zeng L Zhu S Xie Y Liu J Wen Q. Lipid Peroxidation Drives Gasdermin D-Mediated Pyroptosis in Lethal Polymicrobial Sepsis. Cell Host Microbe (2018) 24:97–108 e104. 10.1016/j.chom.2018.05.009 29937272 PMC6043361

[53] Chen R Zhu S Zeng L Wang Q Sheng Y Zhou B. AGER-Mediated Lipid Peroxidation Drives Caspase-11 Inflammasome Activation in Sepsis. Front Immunol (2019) 10:1904. 10.3389/fimmu.2019.01904 31440260 PMC6694796

[54] Zhang L Liu H Jia L Lyu J Sun Y Yu H. Exosomes Mediate Hippocampal and Cortical Neuronal Injury Induced by Hepatic Ischemia-Reperfusion Injury through Activating Pyroptosis in Rats. Oxid Med Cell Longev (2019) 2019:3753485. 10.1155/2019/3753485 31814872 PMC6878784

[55] Liang YB Song PP Zhu YH Xu JM Zhu PZ Liu RR. TREM-1-targeting LP17 attenuates cerebral ischemia-induced neuronal injury by inhibiting oxidative stress and pyroptosis. Biochem Biophys Res Commun (2020) 529:554–61. 10.1016/j.bbrc.2020.05.056 32736673

[56] Li R Zhang LM Sun WB. Erythropoietin rescues primary rat cortical neurons from pyroptosis and apoptosis via Erk1/2-Nrf2/Bach1 signal pathway. Brain Res Bull (2017) 130:236–44. 10.1016/j.brainresbull.2017.01.016 28189515

[57] Gaschler MM Stockwell BR. Lipid peroxidation in cell death. Biochem Biophys Res Commun (2017) 482:419–25. 10.1016/j.bbrc.2016.10.086 PMC5319403 28212725

[58] Yang WS Stockwell BR. Ferroptosis: Death by Lipid Peroxidation. Trends Cell Biol (2016) 26:165–76. 10.1016/j.tcb.2015.10.014 PMC4764384 26653790

[59] Yin H Xu L Porter NA. Free radical lipid peroxidation: mechanisms and analysis. Chem Rev (2011) 111:5944–72. 10.1021/cr200084z 21861450

[60] Zou Y Henry WS Ricq EL Graham ET Phadnis VV Maretich P. Plasticity of ether lipids promotes ferroptosis susceptibility and evasion. Nature (2020) 585:603–8. 10.1038/s41586-020-2732-8 PMC8051864 32939090

[61] Bushnell GW Louie GV Brayer GD. High-resolution three-dimensional structure of horse heart cytochrome c. J Mol Biol (1990) 214:585–95. 10.1016/0022-2836(90)90200-6 2166170

[62] Kobe MJ Neau DB Mitchell CE Bartlett SG Newcomer ME. The structure of human 15-lipoxygenase-2 with a substrate mimic. J Biol Chem (2014) 289:8562–9. 10.1074/jbc.M113.543777 PMC3961679 24497644

[63] Koes DR Baumgartner MP Camacho CJ. Lessons learned in empirical scoring with smina from the CSAR 2011 benchmarking exercise. J Chem Inf Model (2013) 53:1893–904. 10.1021/ci300604z PMC3726561 23379370

[64] Reis A Spickett CM. Chemistry of phospholipid oxidation. Biochim Biophys Acta (BBA)-Biomembranes (2012) 1818:2374–87. 10.1016/j.bbamem.2012.02.002 22342938

[65] Koppenol WH Hider R. Iron and redox cycling. Do’s and don’ts. Free Radical Biol Med (2019) 133:3–10. 10.1016/j.freeradbiomed.2018.09.022 30236787

[66] Stoyanovsky D Tyurina Y Shrivastava I Bahar I Tyurin V Protchenko O. Iron catalysis of lipid peroxidation in ferroptosis: regulated enzymatic or random free radical reaction? Free Radical Biol Med (2019) 133:153–61. 10.1016/j.freeradbiomed.2018.09.008 PMC6555767 30217775

[67] Otterbein LE Soares MP Yamashita K Bach FH. Heme oxygenase-1: unleashing the protective properties of heme. Trends Immunol (2003) 24:449–55. 10.1016/S1471-4906(03)00181-9 12909459

[68] Bochkov VN Oskolkova OV Birukov KG Levonen A-L Binder CJ Stöckl J. Generation and biological activities of oxidized phospholipids. Antioxidants Redox Signaling (2010) 12:1009–59. 10.1089/ars.2009.2597 PMC3121779 19686040

[69] Valko M Jomova K Rhodes CJ Kuča K Musílek K. Redox-and non-redox-metal-induced formation of free radicals and their role in human disease. Arch Toxicol (2016) 90:1–37. 10.1007/s00204-015-1579-5 26343967

[70] Zhao RZ Jiang S Zhang L Yu ZB. Mitochondrial electron transport chain, ROS generation and uncoupling. Int J Mol Med (2019) 44:3–15. 10.3892/ijmm.2019.4188 31115493 PMC6559295

[71] Hayward S Cilliers T Swart P. Lipoxygenases: from isolation to application. Compr Rev Food Sci Food Saf (2017) 16:199–211. 10.1111/1541-4337.12239 33371547

[72] Huttemann M Pecina P Rainbolt M Sanderson TH Kagan VE Samavati L. The multiple functions of cytochrome c and their regulation in life and death decisions of the mammalian cell: From respiration to apoptosis. Mitochondrion (2011) 11:369–81. 10.1016/j.mito.2011.01.010 PMC3075374 21296189

[73] Oellerich S Wackerbarth H Hildebrandt P. Conformational equilibria and dynamics of cytochrome c induced by binding of sodium dodecyl sulfate monomers and micelles. Eur Biophys J (2003) 32:599–613. 10.1007/s00249-003-0306-y 12768249

[74] Kagan VE Bayır HA Belikova NA Kapralov O Tyurina YY Tyurin VA. Cytochrome c/cardiolipin relations in mitochondria: a kiss of death. Free Radical Biol Med (2009) 46:1439–53. 10.1016/j.freeradbiomed.2009.03.004 PMC2732771 19285551

[75] Fadeel B Orrenius S. Apoptosis: a basic biological phenomenon with wide-ranging implications in human disease. J Internal Med (2005) 258:479–517. 10.1111/j.1365-2796.2005.01570.x 16313474

[76] Colombini M. Pore size and properties of channels from mitochondria isolated fromNeurospora crassa. J Membrane Biol (1980) 53:79–84. 10.1007/BF01870576

[77] Dejean LM Martinez-Caballero S Guo L Hughes C Teijido O Ducret T. Oligomeric Bax is a component of the putative cytochrome c release channel MAC, mitochondrial apoptosis-induced channel. Mol Biol Cell (2005) 16:2424–32. 10.1091/mbc.e04-12-1111 PMC1087246 15772159

[78] Kim I Rodriguez-Enriquez S Lemasters JJ. Selective degradation of mitochondria by mitophagy. Arch Biochem biophysics (2007) 462:245–53. 10.1016/j.abb.2007.03.034 PMC2756107 17475204

[79] Chu CT Ji J Dagda RK Jiang JF Tyurina YY Kapralov AA. Cardiolipin externalization to the outer mitochondrial membrane acts as an elimination signal for mitophagy in neuronal cells. Nat Cell Biol (2013) 15:1197–205. 10.1038/ncb2837 PMC3806088 24036476

[80] Kagan VE Tyurina YY Tyurin VA Mohammadyani D Angeli JP Baranov SV. Cardiolipin signaling mechanisms: collapse of asymmetry and oxidation. Antioxid Redox Signal (2015) 22:1667–80. 10.1089/ars.2014.6219 PMC4486147 25566681

[81] Kagan VE Bayır H Tyurina YY Bolevich SB Maguire JJ Fadeel B. Elimination of the unnecessary: intra-and extracellular signaling by anionic phospholipids. Biochem Biophys Res Commun (2017) 482:482–90. 10.1016/j.bbrc.2016.11.005 PMC5319735 28212735

[82] Basova LV Kurnikov IV Wang L Ritov VB Belikova NA Vlasova II. Cardiolipin switch in mitochondria: shutting off the reduction of cytochrome c and turning on the peroxidase activity. Biochemistry (2007) 46:3423–34. 10.1021/bi061854k PMC3356783 17319652

[83] Tuominen EK Wallace CJ Kinnunen PK. Phospholipid-cytochrome c interaction evidence for the extended lipid anchorage. J Biol Chem (2002) 277:8822–6. 10.1074/jbc.M200056200 11781329

[84] Li M Mandal A Tyurin VA DeLucia M Ahn J Kagan VE. Surface-binding to cardiolipin nanodomains triggers cytochrome c pro-apoptotic peroxidase activity via localized dynamics. Structure (2019) 27:806–815. e804. 10.1016/j.str.2019.02.007 30879887 PMC6615723

[85] Kapralov AA Yanamala N Tyurina YY Castro L Samhan-Arias A Vladimirov YA. Topography of tyrosine residues and their involvement in peroxidation of polyunsaturated cardiolipin in cytochrome c/cardiolipin peroxidase complexes. Biochim Biophys Acta (BBA)-Biomembranes (2011) 1808:2147–55. 10.1016/j.bbamem.2011.04.009 PMC3321730 21550335

[86] Vlasova II. Peroxidase activity of human hemoproteins: keeping the fire under control. Molecules (2018) 23:2561. 10.3390/molecules23102561 PMC6222727 30297621

[87] Belikova NA Tyurina YY Borisenko G Tyurin V Samhan-Arias AK Yanamala N. Heterolytic reduction of fatty acid hydroperoxides by cytochrome c/cardiolipin complexes: antioxidant function in mitochondria. J Am Chem Soc (2009) 131:11288–9. 10.1021/ja904343c 19627079

[88] Atkinson J Kapralov AA Yanamala N Tyurina YY Amoscato AA Pearce L. A mitochondria-targeted inhibitor of cytochrome c peroxidase mitigates radiation-induced death. Nat Commun (2011) 2:1–9. 10.1038/ncomms1499 PMC3557495 21988913

[89] Stoyanovsky DA Vlasova II Belikova NA Kapralov A Tyurin V Kagan VE. Activation of NO donors in mitochondria: Peroxidase metabolism of (2-hydroxyamino-vinyl)-triphenyl-phosphonium by cytochrome c releases NO and protects cells against apoptosis. FEBS Lett (2008) 582:725–8. 10.1016/j.febslet.2008.01.047 PMC2275320 18258194

[90] Stockwell BR Angeli JPF Bayir H Bush AI Conrad M Dixon SJ. Ferroptosis: a regulated cell death nexus linking metabolism, redox biology, and disease. Cell (2017) 171:273–85. 10.1016/j.cell.2017.09.021 PMC5685180 28985560

[91] Gaffney BJ. Connecting lipoxygenase function to structure by electron paramagnetic resonance. Accounts Chem Res (2014) 47:3588–95. 10.1021/ar500290r PMC4270396 25341190

[92] Newcomer ME Brash AR. The structural basis for specificity in lipoxygenase catalysis. Protein Sci (2015) 24:298–309. 10.1002/pro.2626 25524168 PMC4353356

[93] Suardíaz R Masgrau L Lluch JM González-Lafont AN. An insight into the regiospecificity of linoleic acid peroxidation catalyzed by mammalian 15-lipoxygenases. J Phys Chem B (2013) 117:3747–54. 10.1021/jp312747q 23496802

[94] Ivanov I Kuhn H Heydeck D. Structural and functional biology of arachidonic acid 15-lipoxygenase-1 (ALOX15). Gene (2015) 573:1–32. 10.1016/j.gene.2015.07.073 26216303 PMC6728142

[95] Kühn H Borchert A. Regulation of enzymatic lipid peroxidation: the interplay of peroxidizing and peroxide reducing enzymes. Free Radical Biol Med (2002) 33:154–72. 10.1016/S0891-5849(02)00855-9 12106812

[96] O’Donnell VB Aldrovandi M Murphy RC Krönke G. Enzymatically oxidized phospholipids assume center stage as essential regulators of innate immunity and cell death. Sci Signal (2019) 12:eaau2293. 10.1126/scisignal.aau2293 30914483

[97] Shah R Shchepinov MS Pratt DA. Resolving the Role of Lipoxygenases in the Initiation and Execution of Ferroptosis. ACS Cent Sci (2018) 4:387–96. 10.1021/acscentsci.7b00589 PMC5879472 29632885

[98] Anthonymuthu TS Tyurina YY Sun WY Mikulska-Ruminska K Shrivastava IH Tyurin VA. Resolving the paradox of ferroptotic cell death: Ferrostatin-1 binds to 15LOX/PEBP1 complex, suppresses generation of peroxidized ETE-PE, and protects against ferroptosis. Redox Biol (2020) 38:101744. 10.1016/j.redox.2020.101744 33126055 PMC7596334

[99] Anthonymuthu TS Kenny EM Shrivastava I Tyurina YY Hier ZE Ting H-C. Empowerment of 15-lipoxygenase catalytic competence in selective oxidation of membrane ETE-PE to ferroptotic death signals, HpETE-PE. J Am Chem Soc (2018) 140:17835–9. 10.1021/jacs.8b09913 PMC6622169 30525572

[100] Wenzel SE Tyurina YY Zhao J St Croix CM Dar HH Mao G. PEBP1 wardens ferroptosis by enabling lipoxygenase generation of lipid death signals. Cell (2017) 171:628–641. e626. 10.1016/j.cell.2017.09.044 29053969 PMC5683852

[101] Tyurina YY Shrivastava I Tyurin VA Mao G Dar HH Watkins S. “Only a Life Lived for Others Is Worth Living”: Redox Signaling by Oxygenated Phospholipids in Cell Fate Decisions. Antioxid Redox Signal (2018) 29:1333–58. 10.1089/ars.2017.7124 PMC6157439 28835115

[102] Bayır H Anthonymuthu TS Tyurina YY Patel SJ Amoscato AA Lamade AM. Achieving Life through Death: Redox Biology of Lipid Peroxidation in Ferroptosis. Cell Chem Biol (2020). 10.1016/j.chembiol.2020.03.014 PMC7218794 32275865

[103] Zou Y Li H Graham ET Deik AA Eaton JK Wang W. Cytochrome P450 oxidoreductase contributes to phospholipid peroxidation in ferroptosis. Nat Chem Biol (2020) 16:302–9. 10.1038/s41589-020-0472-6 PMC7353921 32080622

[104] Winterbourn CC. The biological chemistry of hydrogen peroxide. In: Methods in enzymology, Vol. 528. Elsevier (2013). p. 3–25. 10.1016/B978-0-12-405881-1.00001-X 23849856

[105] Ayala A Munoz MF Arguelles S. Lipid peroxidation: production, metabolism, and signaling mechanisms of malondialdehyde and 4-hydroxy-2-nonenal. Oxid Med Cell Longev (2014) 2014:360438. 10.1155/2014/360438 24999379 PMC4066722

[106] Spickett CM Pitt AR. Modification of proteins by reactive lipid oxidation products and biochemical effects of lipoxidation. Essays Biochem (2020) 64:19–31. 10.1042/EBC20190058 31867621

[107] Pizzimenti S Ciamporcero E Daga M Pettazzoni P Arcaro A Cetrangolo G. Interaction of aldehydes derived from lipid peroxidation and membrane proteins. Front Physiol (2013) 4:242. 10.3389/fphys.2013.00242 24027536 PMC3761222

[108] Agmon E Solon J Bassereau P Stockwell BR. Modeling the effects of lipid peroxidation during ferroptosis on membrane properties. Sci Rep (2018) 8:5155. 10.1038/s41598-018-23408-0 29581451 PMC5979948

[109] Itri R Junqueira HC Mertins O Baptista MS. Membrane changes under oxidative stress: the impact of oxidized lipids. Biophys Rev (2014) 6:47–61. 10.1007/s12551-013-0128-9 28509959 PMC5425709

[110] Tyurina YY Poloyac SM Tyurin VA Kapralov AA Jiang J Anthonymuthu TS. A mitochondrial pathway for biosynthesis of lipid mediators. Nat Chem (2014) 6:542–52. 10.1038/nchem.1924 PMC4201180 24848241

[111] Jiang J Stoyanovsky DA Belikova NA Tyurina YY Zhao Q Tungekar MA. A mitochondria-targeted triphenylphosphonium-conjugated nitroxide functions as a radioprotector/mitigator. Radiat Res (2009) 172:706–17. 10.1667/RR1729.1 PMC2804962 19929417

[112] Tyurina YY Tyurin VA Epperly MW Greenberger JS Kagan VE. Oxidative lipidomics of gamma-irradiation-induced intestinal injury. Free Radic Biol Med (2008) 44:299–314. 10.1016/j.freeradbiomed.2007.08.021 18215738

[113] Tyurina YY Tyurin VA Kapralova VI Wasserloos K Mosher M Epperly MW. Oxidative lipidomics of gamma-radiation-induced lung injury: mass spectrometric characterization of cardiolipin and phosphatidylserine peroxidation. Radiat Res (2011) 175:610–21. 10.1667/RR2297.1 PMC3321728 21338246

[114] Ji J Kline AE Amoscato A Samhan-Arias AK Sparvero LJ Tyurin VA. Lipidomics identifies cardiolipin oxidation as a mitochondrial target for redox therapy of brain injury. Nat Neurosci (2012) 15:1407–13. 10.1038/nn.3195 PMC3697869 22922784

[115] Chen R Feldstein AE McIntyre TM. Suppression of mitochondrial function by oxidatively truncated phospholipids is reversible, aided by bid, and suppressed by Bcl-XL. J Biol Chem (2009) 284:26297–308. 10.1074/jbc.M109.018978 PMC2785317 19654426

[116] Atkinson J Kapralov AA Yanamala N Tyurina YY Amoscato AA Pearce L. A mitochondria-targeted inhibitor of cytochrome c peroxidase mitigates radiation-induced death. Nat Commun (2011) 2:497. 10.1038/ncomms1499 21988913 PMC3557495

[117] Kagan VE Chu CT Tyurina YY Cheikhi A Bayir H. Cardiolipin asymmetry, oxidation and signaling. Chem Phys Lipids (2014) 179:64–9. 10.1016/j.chemphyslip.2013.11.010 PMC3973441 24300280

[118] Kagan VE Bayir HA Belikova NA Kapralov O Tyurina YY Tyurin VE. Cytochrome c/cardiolipin relations in mitochondria: a kiss of death. Free Radic Biol Med (2009) 46:1439–53. 10.1016/j.freeradbiomed.2009.03.004 PMC2732771 19285551

[119] Kapralov AA Kurnikov IV Vlasova II Belikova NA Tyurin VA Basova LV. The hierarchy of structural transitions induced in cytochrome c by anionic phospholipids determines its peroxidase activation and selective peroxidation during apoptosis in cells. Biochemistry (2007) 46:14232–44. 10.1021/bi701237b 18004876

[120] Tyurina YY Tyurin VA Zhao Q Djukic M Quinn PJ Pitt BR. Oxidation of phosphatidylserine: a mechanism for plasma membrane phospholipid scrambling during apoptosis? Biochem Biophys Res Commun (2004) 324:1059–64. 10.1016/j.bbrc.2004.09.102 15485662

[121] Tyurina YY Basova LV Konduru NV Tyurin VA Potapovich AI Cai P. Nitrosative stress inhibits the aminophospholipid translocase resulting in phosphatidylserine externalization and macrophage engulfment: implications for the resolution of inflammation. J Biol Chem (2007) 282:8498–509. 10.1074/jbc.M606950200 17229723

[122] Savill J Gregory C Haslett C. Cell biology. Eat me or die. Science (2003) 302:1516–7. 10.1126/science.1092533 14645835

[123] Segawa K Nagata S. An Apoptotic ‘Eat Me’ Signal: Phosphatidylserine Exposure. Trends Cell Biol (2015) 25:639–50. 10.1016/j.tcb.2015.08.003 26437594

[124] Tyurin VA Balasubramanian K Winnica D Tyurina YY Vikulina AS He RR. Oxidatively modified phosphatidylserines on the surface of apoptotic cells are essential phagocytic ‘eat-me’ signals: cleavage and inhibition of phagocytosis by Lp-PLA2. Cell Death differentiation (2014) 21:825–35. 10.1038/cdd.2014.1 PMC3978307 24464221

[125] Tyurina YY Shvedova AA Kawai K Tyurin VA Kommineni C Quinn PJ. Phospholipid signaling in apoptosis: peroxidation and externalization of phosphatidylserine. Toxicology (2000) 148:93–101. 10.1016/S0300-483X(00)00199-2 10962127

[126] Gaschler MM Andia AA Liu H Csuka JM Hurlocker B Vaiana CA. FINO2 initiates ferroptosis through GPX4 inactivation and iron oxidation. Nat Chem Biol (2018) 14:507–15. 10.1038/s41589-018-0031-6 PMC5899674 29610484

[127] Kagan VE Mao G Qu F Angeli JPF Doll S St Croix C. Oxidized arachidonic and adrenic PEs navigate cells to ferroptosis. Nat Chem Biol (2017) 13:81–90. 10.1038/nchembio.2238 27842066 PMC5506843

[128] Cole BK Kuhn NS Green-Mitchell SM Leone KA Raab RM Nadler JL. 12/15-Lipoxygenase signaling in the endoplasmic reticulum stress response. Am J Physiol Endocrinol Metab (2012) 302:E654–665. 10.1152/ajpendo.00373.2011 PMC3311293 22215650

[129] Stoyanovsky DA Tyurina YY Shrivastava I Bahar I Tyurin VA Protchenko O. Iron catalysis of lipid peroxidation in ferroptosis: Regulated enzymatic or random free radical reaction? Free Radic Biol Med (2019) 133:153–61. 10.1016/j.freeradbiomed.2018.09.008 PMC6555767 30217775

[130] Gao M Monian P Pan Q Zhang W Xiang J Jiang X. Ferroptosis is an autophagic cell death process. Cell Res (2016) 26:1021–32. 10.1038/cr.2016.95 PMC5034113 27514700

[131] Zhou B Liu J Kang R Klionsky DJ Kroemer G Tang D. Ferroptosis is a type of autophagy-dependent cell death. Semin Cancer Biol (2020) 66:89–100. 10.1016/j.semcancer.2019.03.002 30880243

[132] Janikiewicz J Szymanski J Malinska D Patalas-Krawczyk P Michalska B Duszynski J. Mitochondria-associated membranes in aging and senescence: structure, function, and dynamics. Cell Death Dis (2018) 9:332. 10.1038/s41419-017-0105-5 29491385 PMC5832430

[133] Lee S Min KT The Interface Between ER. and Mitochondria: Molecular Compositions and Functions. Mol Cells (2018) 41:1000–7. 10.14348/molcells.2018.0438 PMC6315321 30590907

[134] Imai H Matsuoka M Kumagai T Sakamoto T Koumura T. Lipid Peroxidation-Dependent Cell Death Regulated by GPx4 and Ferroptosis. Curr Top Microbiol Immunol (2017) 403:143–70. 10.1007/82_2016_508 28204974

[135] Liang H Yoo SE Na R Walter CA Richardson A Ran Q. Short form glutathione peroxidase 4 is the essential isoform required for survival and somatic mitochondrial functions. J Biol Chem (2009) 284:30836–44. 10.1074/jbc.M109.032839 PMC2781482 19744930

[136] Pallast S Arai K Wang X Lo EH van Leyen K. 12/15-Lipoxygenase targets neuronal mitochondria under oxidative stress. J Neurochem (2009) 111:882–9. 10.1111/j.1471-4159.2009.06379.x PMC2773137 19737346

[137] Vijayvergiya C De Angelis D Walther M Kuhn H Duvoisin RM Smith DH. High-level expression of rabbit 15-lipoxygenase induces collapse of the mitochondrial pH gradient in cell culture. Biochemistry (2004) 43:15296–302. 10.1021/bi048745v 15568822

[138] van der Veen JN Kennelly JP Wan S Vance JE Vance DE Jacobs RL. The critical role of phosphatidylcholine and phosphatidylethanolamine metabolism in health and disease. Biochim Biophys Acta Biomembr (2017) 1859:1558–72. 10.1016/j.bbamem.2017.04.006 28411170

[139] Battaglia AM Chirillo R Aversa I Sacco A Costanzo F Biamonte F. Ferroptosis and Cancer: Mitochondria Meet the “Iron Maiden” Cell Death. Cells (2020) 9. 10.3390/cells9061505 PMC7349567 32575749

[140] Gao M Yi J Zhu J Minikes AM Monian P Thompson CB. Role of Mitochondria in Ferroptosis. Mol Cell (2019) 73:354–363 e353. 10.1016/j.molcel.2018.10.042 30581146 PMC6338496

[141] Wang H Liu C Zhao Y Gao G. Mitochondria regulation in ferroptosis. Eur J Cell Biol (2020) 99:151058. 10.1016/j.ejcb.2019.151058 31810634

[142] Friedmann Angeli JP Schneider M Proneth B Tyurina YY Tyurin VA Hammond VJ. Inactivation of the ferroptosis regulator Gpx4 triggers acute renal failure in mice. Nat Cell Biol (2014) 16:1180–91. 10.1038/ncb3064 PMC4894846 25402683

[143] Krainz T Gaschler MM Lim C Sacher JR Stockwell BR Wipf P. A Mitochondrial-Targeted Nitroxide Is a Potent Inhibitor of Ferroptosis. ACS Cent Sci (2016) 2:653–9. 10.1021/acscentsci.6b00199 PMC5043442 27725964

[144] Gao H Bai Y Jia Y Zhao Y Kang R Tang D. Ferroptosis is a lysosomal cell death process. Biochem Biophys Res Commun (2018) 503:1550–6. 10.1016/j.bbrc.2018.07.078 30031610

[145] Torii S Shintoku R Kubota C Yaegashi M Torii R Sasaki M. An essential role for functional lysosomes in ferroptosis of cancer cells. Biochem J (2016) 473:769–77. 10.1042/BJ20150658 26759376

[146] Magtanong L Ko PJ To M Cao JY Forcina GC Tarangelo A. Exogenous Monounsaturated Fatty Acids Promote a Ferroptosis-Resistant Cell State. Cell Chem Biol (2019) 26:420–32.e429. 10.1016/j.chembiol.2018.11.016 30686757 PMC6430697

[147] Tarangelo A Dixon SJ. Lipid Metabolism and Ferroptosis. (2019). pp. 1–26. 10.1007/978-3-030-26780-3_1

[148] Kim SE Zhang L Ma K Riegman M Chen F Ingold I. Ultrasmall nanoparticles induce ferroptosis in nutrient-deprived cancer cells and suppress tumour growth. Nat Nanotechnol (2016) 11:977–85. 10.1038/nnano.2016.164 PMC5108575 27668796

[149] Linkermann A Skouta R Himmerkus N Mulay SR Dewitz C De Zen F. Synchronized renal tubular cell death involves ferroptosis. Proc Natl Acad Sci USA (2014) 111:16836–41. 10.1073/pnas.1415518111 PMC4250130 25385600

[150] Poursaitidis I Wang X Crighton T Labuschagne C Mason D Cramer SL. Oncogene-Selective Sensitivity to Synchronous Cell Death following Modulation of the Amino Acid Nutrient Cystine. Cell Rep (2017) 18:2547–56. 10.1016/j.celrep.2017.02.054 PMC5368412 28297659

[151] Aryal B Rao VA. Deficiency in Cardiolipin Reduces Doxorubicin-Induced Oxidative Stress and Mitochondrial Damage in Human B-Lymphocytes. PloS One (2016) 11:e0158376. 10.1371/journal.pone.0158376 27434059 PMC4951097

[152] Maguire JJ Tyurina YY Mohammadyani D Kapralov AA Anthonymuthu TS Qu F. Known unknowns of cardiolipin signaling: The best is yet to come. Biochim Biophys Acta Mol Cell Biol Lipids (2017) 1862:8–24. 10.1016/j.bbalip.2016.08.001 27498292 PMC5323096

[153] McClelland LJ Mou TC Jeakins-Cooley ME Sprang SR Bowler BE. Structure of a mitochondrial cytochrome c conformer competent for peroxidase activity. Proc Natl Acad Sci USA (2014) 111:6648–53. 10.1073/pnas.1323828111 PMC4020089 24760830

[154] Bakan A Kapralov AA Bayir H Hu F Kagan VE Bahar I. Inhibition of Peroxidase Activity of Cytochrome c: De Novo Compound Discovery and Validation. Mol Pharmacol (2015) 88:421–7. 10.1124/mol.115.097816 PMC4551054 26078313

[155] Epperly MW Sacher JR Krainz T Zhang X Wipf P Liang M. Effectiveness of Analogs of the GS-Nitroxide, JP4-039, as Total Body Irradiation Mitigators. In Vivo (2017) 31:39–43. 10.21873/invivo.11022 28064218 PMC5267945

[156] Kagan VE Bayir A Bayir H Stoyanovsky D Borisenko GG Tyurina YY. Mitochondria-targeted disruptors and inhibitors of cytochrome c/cardiolipin peroxidase complexes: a new strategy in anti-apoptotic drug discovery. Mol Nutr Food Res (2009) 53:104–14. 10.1002/mnfr.200700402 PMC2659540 18979502

[157] Cao JY Dixon SJ. Mechanisms of ferroptosis. Cell Mol Life Sci (2016) 73:2195–209. 10.1007/s00018-016-2194-1 PMC4887533 27048822

[158] Dodson M Castro-Portuguez R Zhang DD. NRF2 plays a critical role in mitigating lipid peroxidation and ferroptosis. Redox Biol (2019) 23:101107. 10.1016/j.redox.2019.101107 30692038 PMC6859567

[159] Song X Long D. Nrf2 and Ferroptosis: A New Research Direction for Neurodegenerative Diseases. Front Neurosci (2020) 14:267. 10.3389/fnins.2020.00267 32372896 PMC7186402

[160] Abdalkader M Lampinen R Kanninen KM Malm TM Liddell JR. Targeting Nrf2 to Suppress Ferroptosis and Mitochondrial Dysfunction in Neurodegeneration. Front Neurosci (2018) 12:466. 10.3389/fnins.2018.00466 30042655 PMC6048292

[161] Circu ML Aw TY. Glutathione and apoptosis. Free Radic Res (2008) 42:689–706. 10.1080/10715760802317663 18671159 PMC3171829

[162] Aquilano K Baldelli S Ciriolo MR. Glutathione: new roles in redox signaling for an old antioxidant. Front Pharmacol (2014) 5:196. 10.3389/fphar.2014.00196 25206336 PMC4144092

[163] Feng H Stockwell BR. Unsolved mysteries: How does lipid peroxidation cause ferroptosis? PloS Biol (2018) 16:e2006203. 10.1371/journal.pbio.2006203 29795546 PMC5991413

[164] Shimada K Skouta R Kaplan A Yang WS Hayano M Dixon SJ. Global survey of cell death mechanisms reveals metabolic regulation of ferroptosis. Nat Chem Biol (2016) 12:497–503. 10.1038/nchembio.2079 27159577 PMC4920070

[165] Stockwell BR Jiang X Gu W. Emerging Mechanisms and Disease Relevance of Ferroptosis. Trends Cell Biol (2020) 30:478–90. 10.1016/j.tcb.2020.02.009 PMC7230071 32413317

[166] Kagan VE Serbinova EA Safadi A Catudioc JD Packer L. NADPH-dependent inhibition of lipid peroxidation in rat liver microsomes. Biochem Biophys Res Commun (1992) 186:74–80. 10.1016/S0006-291X(05)80777-6 1632795

[167] Maguire JJ Kagan V Ackrell BA Serbinova E Packer L. Succinate-ubiquinone reductase linked recycling of alpha-tocopherol in reconstituted systems and mitochondria: requirement for reduced ubiquinone. Arch Biochem Biophys (1992) 292:47–53. 10.1016/0003-9861(92)90049-3 1727650

[168] Kagan VE Tyurina YY. Recycling and redox cycling of phenolic antioxidants. Ann N Y Acad Sci (1998) 854:425–34. 10.1111/j.1749-6632.1998.tb09921.x 9928449

[169] Kagan VE Packer L. Light-induced generation of vitamin E radicals: assessing vitamin E regeneration. Methods Enzymol (1994) 234:316–20. 10.1016/0076-6879(94)34099-4 7808300

[170] Ross D Siegel D. Functions of NQO1 in Cellular Protection and CoQ10 Metabolism and its Potential Role as a Redox Sensitive Molecular Switch. Front Physiol (2017) 8:595. 10.3389/fphys.2017.00595 28883796 PMC5573868

[171] Villalba JM Navarro F Gómez-Díaz C Arroyo A Bello RI Navas P. Role of cytochrome b5 reductase on the antioxidant function of coenzyme Q in the plasma membrane. Mol Aspects Med (1997) 18 Suppl:S7–13. 10.1016/S0098-2997(97)00015-0 9266501

[172] Paradies G Paradies V De Benedictis V Ruggiero FM Petrosillo G. Functional role of cardiolipin in mitochondrial bioenergetics. Biochim Biophys Acta (BBA) - Bioenergetics (2014) 1837:408–17. 10.1016/j.bbabio.2013.10.006 24183692

[173] Kagan VE Jiang J Huang Z Tyurina YY Desbourdes C Cottet-Rousselle C. NDPK-D (NM23-H4)-mediated externalization of cardiolipin enables elimination of depolarized mitochondria by mitophagy. Cell Death differentiation (2016) 23:1140–51. 10.1038/cdd.2015.160 PMC4946882 26742431

[174] Van Q Liu J Lu B Feingold KR Shi Y Lee RM. Phospholipid scramblase-3 regulates cardiolipin de novo biosynthesis and its resynthesis in growing HeLa cells. Biochem J (2007) 401:103–9. 10.1042/BJ20060373 PMC1698660 16939411

[175] Kooijman EE Swim LA Graber ZT Tyurina YY Bayır H Kagan VE. Magic angle spinning (31)P NMR spectroscopy reveals two essentially identical ionization states for the cardiolipin phosphates in phospholipid liposomes. Biochim Biophys Acta Biomembr (2017) 1859:61–8. 10.1016/j.bbamem.2016.10.013 PMC5362297 27984017

[176] Droghetti E Oellerich S Hildebrandt P Smulevich G. Heme coordination states of unfolded ferrous cytochrome C. Biophys J (2006) 91:3022–31. 10.1529/biophysj.105.079749 PMC1578467 16877519

[177] Barker PD Nerou EP Cheesman MR Thomson AJ de Oliveira P Hill HA. Bis-methionine ligation to heme iron in mutants of cytochrome b562. 1. Spectroscopic and electrochemical characterization of the electronic properties. Biochemistry (1996) 35:13618–26. 10.1021/bi961127x 8885841

[178] Di Marino M Marassi R Santucci R Brunori M Ascoli F. A spectroelectrochemical study of carboxymethylated cytochrome-c. Bioelectrochem Bioenergetics (1987) 17:27–34. 10.1016/0302-4598(87)80004-1

[179] Tsong TY. Ferricytochrome c chain folding measured by the energy transfer of tryptophan 59 to the heme group. Biochemistry (1976) 15:5467–73. 10.1021/bi00670a007 11814

[180] Ardail D Privat JP Egret-Charlier M Levrat C Lerme F Louisot P. Mitochondrial contact sites. Lipid composition and dynamics. J Biol Chem (1990) 265:18797–802. 10.1016/S0021-9258(17)30583-5 2172233

[181] Kagan VE Tyurin VA Jiang J Tyurina YY Ritov VB Amoscato AA. Cytochrome c acts as a cardiolipin oxygenase required for release of proapoptotic factors. Nat Chem Biol (2005) 1:223–32. 10.1038/nchembio727 16408039

[182] Liu G-Y Moon SH Jenkins CM Li M Sims HF Guan S. The phospholipase iPLA2γ is a major mediator releasing oxidized aliphatic chains from cardiolipin, integrating mitochondrial bioenergetics and signaling. J Biol Chem (2017) 292:10672–84. 10.1074/jbc.M117.783068 PMC5481572 28442572

[183] Dennis EA Norris PC. Eicosanoid storm in infection and inflammation. Nat Rev Immunol (2015) 15:511. 10.1038/nri3859 26139350 PMC4606863

[184] Lamade AM Kenny EM Anthonymuthu TS Soysal E Clark RSB Kagan VE. Aiming for the target: Mitochondrial drug delivery in traumatic brain injury. Neuropharmacology (2019) 145:209–19. 10.1016/j.neuropharm.2018.07.014 PMC6309489 30009835

[185] Tyurina YY Tungekar MA Jung MY Tyurin VA Greenberger JS Stoyanovsky DA. Mitochondria targeting of non-peroxidizable triphenylphosphonium conjugated oleic acid protects mouse embryonic cells against apoptosis: role of cardiolipin remodeling. FEBS Lett (2012) 586:235–41. 10.1016/j.febslet.2011.12.016 PMC3273856 22210054

[186] Shah R Shchepinov MS Pratt DA. Resolving the Role of Lipoxygenases in the Initiation and Execution of Ferroptosis. ACS Cent Sci (2018) 4:387–96. 10.1021/acscentsci.7b00589 PMC5879472 29632885

[187] Shah R Farmer LA Zilka O Van Kessel ATM Pratt DA. Beyond DPPH: Use of Fluorescence-Enabled Inhibited Autoxidation to Predict Oxidative Cell Death Rescue. Cell Chem Biol (2019) 26:1594–607.e1597. 10.1016/j.chembiol.2019.09.007 31564533

[188] Anthonymuthu TS Kenny EM Shrivastava I Tyurina YY Hier ZE Ting H-C. Empowerment of 15-Lipoxygenase Catalytic Competence in Selective Oxidation of Membrane ETE-PE to Ferroptotic Death Signals, HpETE-PE. J Am Chem Soc (2018) 140:17835–9. 10.1021/jacs.8b09913 PMC6622169 30525572

[189] Tyurina YY St Croix CM Watkins SC Watson AM Epperly MW Anthonymuthu TS. Redox (phospho)lipidomics of signaling in inflammation and programmed cell death. J Leukoc Biol (2019) 106:57–81. 10.1002/JLB.3MIR0119-004RR 31071242 PMC6626990

